# Transposon mutagenesis identifies cooperating genetic drivers during keratinocyte transformation and cutaneous squamous cell carcinoma progression

**DOI:** 10.1371/journal.pgen.1009094

**Published:** 2021-08-16

**Authors:** Aziz Aiderus, Justin Y. Newberg, Liliana Guzman-Rojas, Ana M. Contreras-Sandoval, Amanda L. Meshey, Devin J. Jones, Felipe Amaya-Manzanares, Roberto Rangel, Jerrold M. Ward, Song-Choon Lee, Kenneth Hon-Kim Ban, Keith Rogers, Susan M. Rogers, Luxmanan Selvanesan, Leslie A. McNoe, Neal G. Copeland, Nancy A. Jenkins, Kenneth Y. Tsai, Michael A. Black, Karen M. Mann, Michael B. Mann

**Affiliations:** 1 Department of Molecular Oncology, Moffitt Cancer Center & Research Institute, Tampa, Florida, United States of America; 2 Cancer Research Program, Houston Methodist Research Institute, Houston, Texas, United States of America; 3 Institute of Molecular and Cell Biology, Agency for Science, Technology and Research (A*STAR), Biopolis, Singapore, Republic of Singapore; 4 Centre for Translational Cancer Research, Department of Biochemistry, University of Otago, Dunedin, New Zealand; 5 Departments of Anatomic Pathology & Tumor Biology, Moffitt Cancer Center & Research Institute, Tampa, Florida, United States of America; 6 Donald A. Adam Melanoma and Skin Cancer Research Center of Excellence, Moffitt Cancer Center & Research Institute, Tampa, Florida, United States of America; 7 Department of Oncologic Sciences, Morsani College of Medicine, University of South Florida, Tampa, Florida, United States of America; 8 Departments of Gastrointestinal Oncology & Malignant Hematology, Moffitt Cancer Center & Research Institute, Tampa, Florida, United States of America; 9 Cancer Biology and Evolution Program, Moffitt Cancer Center & Research Institute, Tampa, Florida, United States of America; 10 Department of Cutaneous Oncology, Moffitt Cancer Center & Research Institute, Tampa, Florida, United States of America; Seattle Children’s Research Institute, UNITED STATES

## Abstract

The systematic identification of genetic events driving cellular transformation and tumor progression in the absence of a highly recurrent oncogenic driver mutation is a challenge in cutaneous oncology. In cutaneous squamous cell carcinoma (cuSCC), the high UV-induced mutational burden poses a hurdle to achieve a complete molecular landscape of this disease. Here, we utilized the *Sleeping Beauty* transposon mutagenesis system to statistically define drivers of keratinocyte transformation and cuSCC progression *in vivo* in the absence of UV-IR, and identified both known tumor suppressor genes and novel oncogenic drivers of cuSCC. Functional analysis confirms an oncogenic role for the *ZMIZ* genes, and tumor suppressive roles for *KMT2C*, *CREBBP* and *NCOA2*, in the initiation or progression of human cuSCC. Taken together, our *in vivo* screen demonstrates an extremely heterogeneous genetic landscape of cuSCC initiation and progression, which can be harnessed to better understand skin oncogenic etiology and prioritize therapeutic candidates.

## Author summary

Non-melanoma skin cancers, the most common cancers in the US, are caused by UV skin exposure. Nearly 1 million cases of cutaneous squamous cell carcinoma (cuSCC) are diagnosed in the US each year. While most cuSCCs are highly treatable, more than twice as many individuals die from this disease as from melanoma. The high burden of UV-induced DNA damage in human skin poses a challenge for identifying initiating and cooperating mutations that promote cuSCC development and for defining potential therapeutic targets. Here, we describe a genetic screen in mice using a DNA transposon system to mutagenize the genome of keratinocytes and drive squamous cell carcinoma in the absence of UV. By sequencing where the transposons selectively integrated in the genomes of normal skin, skin with pre-cancerous lesions and skin with fully developed cuSCCs from our mouse model, we were able to identify frequently mutated genes likely important for this disease. Our analysis also defined cooperation between sets of genes not previously appreciated in cuSCC. Our mouse model and ensuing data provide a framework for understanding the genetics of cuSCC and for defining the molecular changes that may lead to the future therapies for patients.

## Introduction

Cutaneous squamous cell carcinoma (cuSCC) is the second most common cancer in man, with approximately one million cases diagnosed annually in the United States. Although the majority of cuSCC are considered a low-risk neoplasm, up to 5% of high-risk cuSCCs are locally or distantly invasive and carry a poor prognosis due to a lack of biomarkers, therapeutic targets, or FDA-approved molecularly targeted therapies. This represents a substantial unmet need for approximately 50,000 patients per year with high-risk cuSCC, and an opportunity to identify new therapeutic modalities that could improve disease outcomes. All non-viral associated skin cancers are thought to require multiple cooperating mutations that deregulate distinct signaling pathways to initiate and progress the multi-step transformation of normal cells into a clinically significant neoplasm. Indeed, identifying cooperating mutations that drive malignant transformation is a prerequisite for developing better combinatorial therapies for managing and treating skin cancers. Most skin cancers, including cuSCC [1, 2], have the highest mutation rates among human cancers due to ultraviolet irradiation (UV-IR) induced damage from chronic, intermittent sun exposure. Thus, using human cancer sequencing data alone, with some of the highest mutational burdens of any cancer, poses challenges to identify cooperating, low-penetrant mutations that lead to cancer progression. This presents a need to develop *in vivo* model systems to help identify and prioritize novel cooperating candidate cancer drivers for keratinocyte transformation and subsequent progression to invasive cuSCC.

*Sleeping Beauty* (SB) insertional mutagenesis [3] is a powerful tool used to perform genome-wide forward genetic screens in laboratory mice for cancer gene discovery [4–14] in animal models of both hematopoietic and solid tumors [12, 15]. The SB system employs a DNA transposon that is mobilized throughout the genome by a conditional Cre-induced transposase expressed in trans that binds the inverted repeats at the end of the transposon, initiates DNA double-strand breaks and reintegrates the transposon randomly at TA-dinucleotides (reviewed in [5]). The transposon itself contains a minimal internal promoter and splice-donor (SD) and bidirectional splice-acceptor (SA) sequences, such that the SB transposons can either activate proto-oncogenes or inactivate tumor suppressor genes, and identify early cancer progression drivers that cooperate to initiate tumors [11, 14] and potentially drive metastasis [16, 17]. Importantly, SB insertions induce changes in gene expression, thus providing epigenetic information not easily obtained from carcinogenesis mouse models using chemical [18] or chronic UV irradiation [19, 20] or from limited patient samples. We demonstrate that SB mobilization of a low-copy T2/Onc3 transposon allele is sufficient to induce and progress a variety of cancers *in vivo*. Here, we report our efforts for cancer gene discovery in skin tumors. Using high-throughput sequencing approaches [9, 11] and our SB Driver Analysis [21] statistical framework, we profiled genome-wide SB mutations from cuKA and cuSCC and defined recurrently mutated, statistically significant candidate cancer drivers (CCDs) from bulk cuSCC tumors and from normal keratinocytes and early stage tumors, identifying both known tumor suppressor genes and novel oncogenic drivers. We further prioritized oncogene and tumor suppressor candidates and provide *in vitro* and *in vivo* functional evidence for the roles of these genes in the initiation and progression of cuSCC. Taken together, our efforts provide a systematic evolutionary landscape of cuSCC genesis, and highlights key pathways promoting disease that are potential therapeutic targets.

## Results

### Candidate cancer driver discovery in SB-driven keratinocyte cancer models

**Fig 1:**
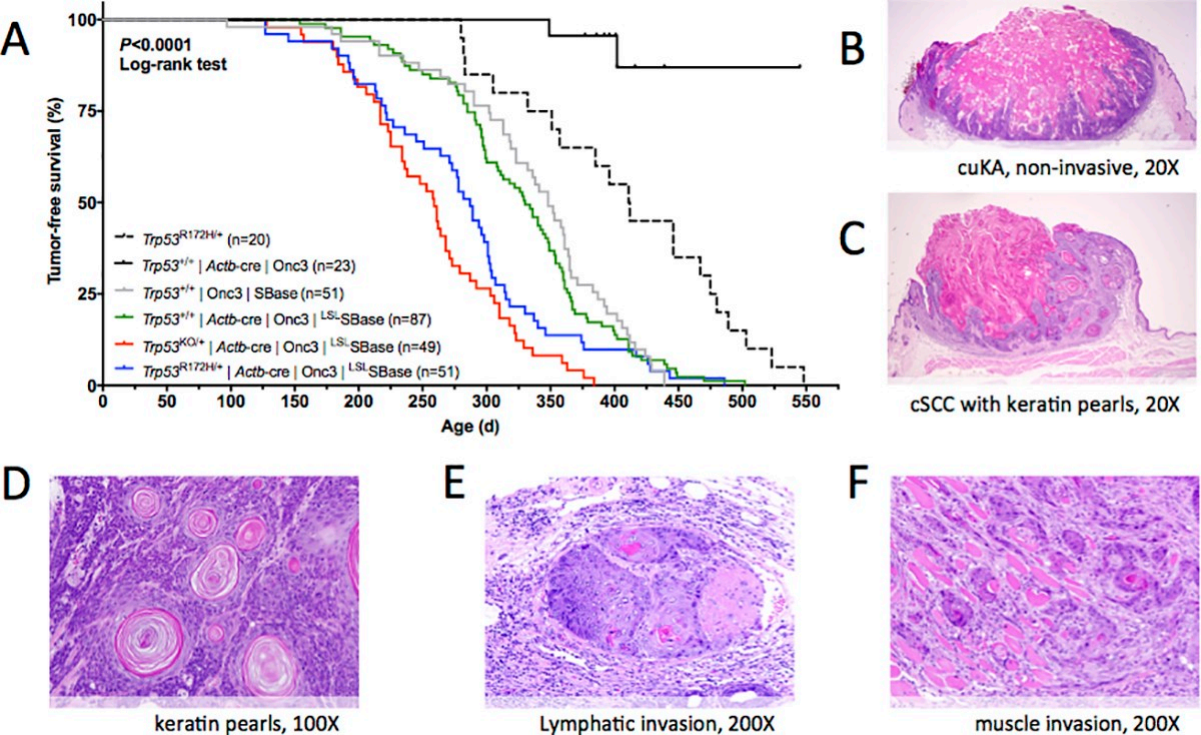
Whole-body SB transposon mutagenesis drives a diverse tumor spectrum in *Trp53* wild type and mutant mice. (**A**) Kaplan-Meier survival curves comparing experimental SB and non-SB control mouse cohorts (log-rank test, *P* < 0.0001). Wild type *Trp53* cohorts with SB-Onc3 mobilized by a constitutively active *Rosa26*-SBase allele (Gray line, reported in [7]) do not differ significantly from SB-Onc3 mice mobilized by a conditionally activated *Rosa26*-LSL-SBase allele to the *Rosa26*-1lox-SBase allele by whole body *Cre* expression (log-rank test, *P*>0.05). Mice in all SB-Onc3 cohorts developed solid tumors, including cutaneous squamous cell carcinoma (cuSCC) and hepatocellular adenoma (HCA), but *Trp53*^+/−^ (red line) and *Trp53*^R172H/+^ (blue line) mice had significantly decreased survival compared to the wild-type (WT) cohort (Green line, *P*<0.0001, log-rank). Non-SB control *Trp53*^R172H/+^ mice (dashed black line) had significantly decreased survival compared to the WT cohort (solid black line, *P*<0.0001, log-rank). (**B-C**) Histology and tumor classification from sections of skin masses stained with hematoxylin and eosin. (**B**) Early-stage, non-invasive mass with cutaneous keratoacanthoma-like morphology (cuKA) in SB-Onc3|*Trp53*^+/+^ mouse (20×). (**C**) Invasive mass with cuSCC displaying keratin pearl morphology in SB-Onc3|*Trp53*^KO/+^ mouse (20×). (**D**) cuSCC keratin pearls in SB-Onc3|*Trp53*^+/+^ mouse (100×). (**E**) cuSCC displaying lymphatic invasion in SB-Onc3|*Trp53*^R172H/+^ mouse (200×). (**F**) cuSCC displaying muscle invasion in SB-Onc3|*Trp53*^+/+^ mouse (200×).

We performed a forward genetic screen using SB insertional mutagenesis to define the genes that cooperate with *Trp53* mutant alleles to drive tumorigenesis. Mice carrying either a conditional *Trp53* null (*Trp53*^*KO/+*^) [22] or recurrent point mutant allele (*Trp53*^*R172H/+*^) [23] and *Actb-Cre* transgene [24] were crossed to Sleeping Beauty mice double homozygous for a low-copy, bi-functional T2/Onc3 transposon (12740) and an inducible SB transposase [7] (S1 **Fig**). The resulting progeny, referred to as SB|Onc3 mice, exhibited whole-body mutagenesis and succumbed to a wide variety of solid and hematopoietic tumor types with variable penetrance (S1 **Table**). The presence of a *Trp53* mutant allele accelerated tumor progression and significantly reduced tumor-free survival relative to *Trp53* wild-type littermate controls (*P*<0.0001, **Fig 1A**). The SB|Onc3 mice with wildtype *Trp53* exhibited a disease penetrance and latency similar to T2/Onc3 cohorts produced with constitutively active SBase [7]. SB|*Trp53*^*KO/+*^ mice were the earliest to reach endpoint, but had the lowest overall tumor burden, presumably because these mice had the highest proportion of hematopoietic disease. SB|*Trp53*^*R172H/+*^ mice reached endpoint earlier than SB|*Trp53*^*+/+*^ mice and had the widest spectrum of solid tumors. The most prevalent of the more than 20 solid tumor types observed were early-stage cutaneous keratoacanthoma (cuKA) (**Fig 1B**) and well-differentiated cutaneous squamous cell carcinoma (cuSCC) (**Fig 1C and 1F**). Importantly, these cuSCC/cuKAs were absent in mice with mutant *Trp53* lacking SB, strongly suggesting SB mutagenesis drives initiation of cellular transformation and subsequent cancer progression of cuSCC *in vivo*.

### SBCapSeq defines recurrent transposon events in normal, premalignant, and tumor skin cell genomes

**Fig 2:**
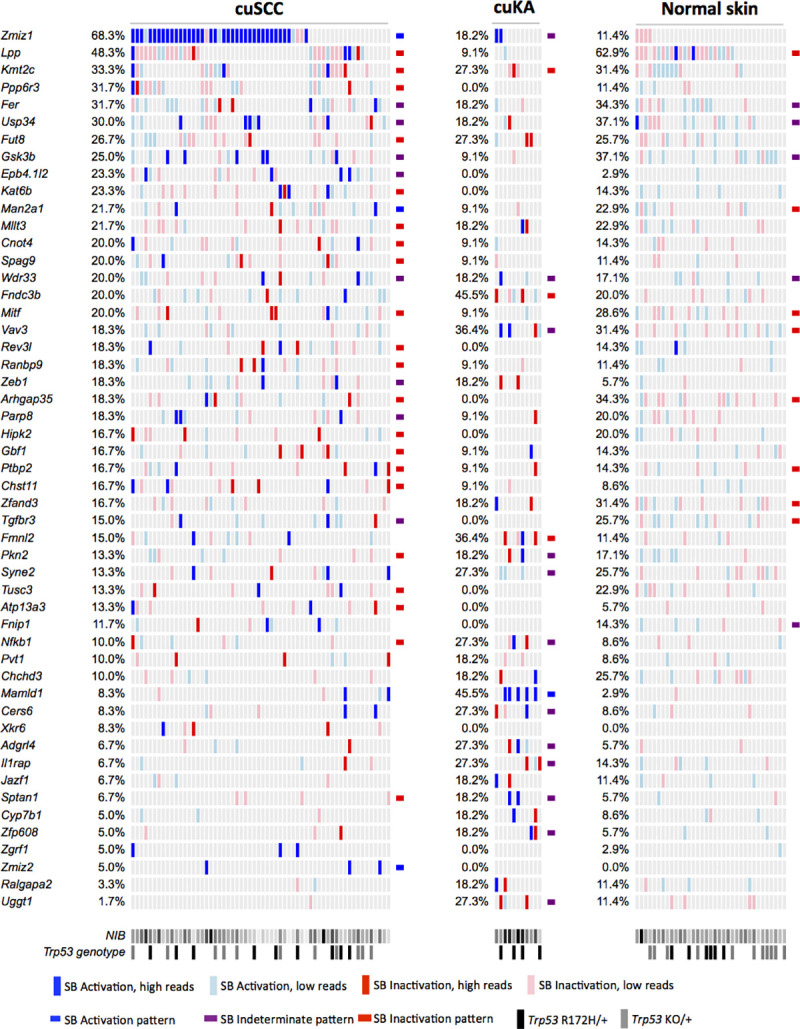
Landscape of trunk drivers mutated during SB-induced keratinocyte transformation and cuSCC progression using SBCapSeq. SB Trunk Driver Analysis, with read depth cutoff of 300, was performed using SBCapSeq insertion data from the SB|cuSCC and SB|cuKA tumor cohorts (the normal skin cohort did not identify any significant trunk drivers). Drivers with significant family-wise error rate (FWER) adjusted *P*-values were merged into a single list and plotted from each of the cuSCC, cuKA and normal skin specimens by occurrence within the cuSCC cohort. Vertical colored bars represent genomes (columns) with SB insertion events that occur within the same (sense, blue) or opposite (anti-sense, red) DNA strand relative to transcription of a driver gene (row). High read depth sites (>299 reads supporting an SB insertion event, blue or red) are distinguished from low read depth sites (<300 reads supporting an SB insertion event, light blue or pink) to denote clonal and sub-clonal SB insertion events, respectively (**S1, S2, and S3 Data**). Driver gene classifications (indeterminate, activating or Inactivating) are shown as a bar at the end of a gene on a cohort specific manner where calculation was possible. NIB, normalized insertion burden from highest (black) to lowest (light gray). *Trp53* genotypes: *Trp53* ^+/+^ (white), *Trp53* ^R172H/+^ (black), and *Trp53* ^KO/+^ (gray).

To investigate the genetic events underlying the SB-driven keratinocyte transformation and cuSCC progression in wildtype and *Trp53* mutant mice, we sequenced SB insertions sites from 60 histologically confirmed cuSCCs, 11 cuKA and 32 normal skin genomes (S2 **Table**) using an enhanced version of SB Capture Sequencing (SBCapSeq) from our previously published workflow [9] that is optimized for liquid-phase capture of transposons from both normal and tumor specimen genomes. Using the SBCapSeq bioinformatics workflow [9] and gene-guided SB Driver Analysis statistical framework [21], we identified 11,113,694 reads at 59,337 non-redundant transposon insertion sites from cuSCCs which were used to statistically define 1,333 candidate cancer driver genes (S3 **Table**). Nearly all CCDs were identified across all cohorts, suggesting that their role in driving cuSCC is independent of *Trp53* mutation. Stringent filtering of the SB insertions to include only events with the highest sequencing read depths defines trunk driver alterations predicted to be under positive-selection within the clonally expanding tumors, where they participate in either initiation or early progression of cellular transformation [9, 11]. To identify CCDs that drive keratinocyte transformation, we selected the SB insertion sites with the highest number of sequencing reads to statistically define trunk drivers as previously described [21, 25], reasoning that tumor initiating insertions would show the highest frequency in tumor cells (see Methods). We identified 86 trunk drivers (S4 **Table**). The top two most significant trunk drivers in cuSCC genomes were the paralogous transcriptional co-factors *Zmiz1* and *Zmiz2*, which are collectively and mutually exclusively mutated in 71% of end-stage SB|cuSCC genomes (S2 **Fig**). Using the same methodology, we next analyzed cuKA specimens harvested either from mice with cuSCC or from an independent age- and genotype-matched cohort and identified 2,085,280 reads at 6,727 non-redundant transposon insertion sites, defining 192 candidate cancer driver genes (S5 **Table**) and 10 trunk drivers (S6 **Table**). Finally, we identified 2,910,945 reads at 47,537 non-redundant transposon insertion sites from normal skin specimens with statistically defined enrichment in 547 genes (S7 **Table**). No trunk drivers were identified. The histologically normal skin genomes from mice with SB mobilization had a mean of 1,320 insertion events (range 1–3,857). Notably, fewer than 1% of the insertions had read depths >300 reads per site (range 1–2,297), which is one order of magnitude less than 23.7% of cuKA genomes or 8.5% of cuSCC genomes with detected insertion events at >300 reads per specimen (cuKA range 1–19,798 and cuSCC range 1–35,809). An oncoprint illustrates the read-depth and insertion patterns that define the trunk drivers in cuSCC and cuKA (**Fig 2**), comparing the incidence of selected insertions in these same drivers in normal skin. Together, these data strongly suggest that clonal selection of recurrent, high-read depth SB insertion sites is featured prominently within the cuSCC and cuKA genomes and almost entirely missing within the normal skin genomes.

Further evidence for clonal selection associated with disease progression was provided by technical replication of SBCapSeq libraries, demonstrating excellent biological reproducibility (sequencing of two independent library preparations from single isolation of genomic DNA) of cuSCC genomes (**S3A and S3B Fig**) and no reproducibility in normal skin genomes (**S3C and S3D Fig**), illustrating the absence of clonal expansion in the normal samples despite the high insertion burden. Importantly, replicate sequencing of a single library preparation from two normal skin samples demonstrated excellent technical reproducibility (**S3E and S3F Fig**), further supporting the robustness of our methodology to detect clonal selection. These observations are consistent with the large numbers of mutations observed during chronic UV-IR exposure in human eyelid [26] and SKH hairless mouse [19, 20] skin and, together with our SB data, indicates that keratinocytes within phenotypically normal skin can tolerate high background mutagenic insults that may act as a mutational reservoir prior to clonal selection of cooperating driver mutations that permit frank outgrown of cuSCC *in vivo*. This is likely the reason that no trunk drivers could be identified from the normal skin specimen genomes. The quantitative, stage-dependent insertional mutation burdens derived from SBCapSeq in cutaneous specimens reveal incredible levels of tumor heterogeneity in SB-driven keratinocyte transformation and progression to cuSCC.

### cuSCC demonstrate clonal and sub-clonal intratumor heterogeneity

**Fig 3:**
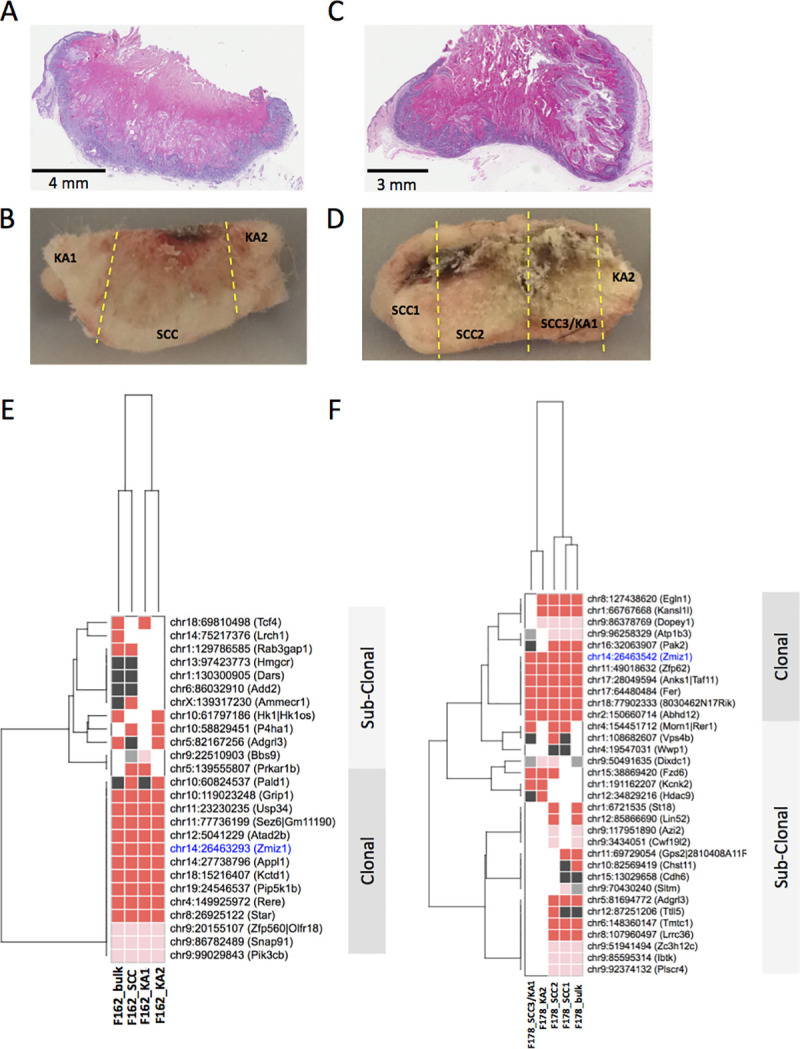
Multi-region SBCapSeq analysis of dual histology keratinocyte-derived skin masses containing distinct cuKA and cuSCC histological regions. Two individual mice displayed lesions with both cuKA and cuSCC histologies suggestive of clonal evolution of cuSCC differentiation within cuKA masses. Aperio slide scans of H&E stained FFPE specimens and the adjacent flash frozen tissue for specimen 1 (**A-B**) and specimen 2 (**C-D**) prior to sectioning for genomic DNA isolation. Yellow dotted lines demarcate the distinct lesions within each section that were sampled for sequencing SB insertions. Hierarchical two-dimensional clustering (Hamming distance with the Ward method) of recurrent genic SBCapSeq insertion data for specimen 1 (**E**) and specimen 2 (**F**) demonstrates trunk (common to all samples) and sub-clonal, lesion-specific SB insertions (**S4 Data**). Genomic DNA was isolated from adjacent serial sections and included a “bulk” reference consisting of a cross-section of entire mass, and three or four regions dissected from histologically distinct regions.

We identified four skin masses with histologically continuous but distinct regions of cuKA and cuSCC, suggesting for the first time that cuKA may progress directly to invasive cuSCC *in vivo*. Examples of gross and histological sections are shown in **Fig 3A, 3B, 3C**, and **3D**. We performed SBCapSeq on representative, histologically distinct regions to investigate intratumor heterogeneity and to gain insight into the drivers likely to mediate cuKA to cuSCC progression. Regional sequencing gave an overall lower sequencing depth than our bulk tumor sequencing. Therefore, we adjusted the minimum read-depth threshold to 200 reads for defining clonal insertions. For several loci, we identified insertions at the same nucleotide address across tumor regions from both cuKA and cuSCC in the same mouse, indicating clonal origins of these lesions (S8 **Table**). Hierarchical two-dimensional clustering of recurrent genic SBCapSeq insertion data from four skin masses containing distinct cuKA and cuSCC regions is shown in **Fig 3E**. Invariably, clonal insertions into *Zmiz1* were identified across each section of all 4 masses at three different TA-dinucleotide addresses, suggesting that *Zmiz1* was under positive selection within the nascent transformed cells that gave rise to the distinct cuKA and cuSCC features. We also found evidence for sub-clonal insertion events identified at the same nucleotide address in some but not all regions within the same tumor, demonstrating the evolution of the tumor. Some genes with sub-clonal insertion events with read depths greater than 200 (see Methods) were defined as drivers in the bulk population, including *Tcf4* and *Pak2*, *while other genes*, *such as Egr2* and *Rab3gap1*, were not (**Figs 3F** and **S4**). These data provide evidence for a common keratinocyte progenitor that acquires SB insertions in genes that promote cuKA and are maintained in cuSCC. This analysis also highlights the intra- and inter-tumor heterogeneity of insertions in SB cuSCC tumors and importantly demonstrates that SB insertion profiles can be used to trace the evolution of cuKA to cuSCC using both the insertion nucleotide address and read depth to define clonal relationships.

### Comparative oncogenomic meta-analysis

One hundred-seven of the SB candidate Trunk driver genes are direct orthologs of human cancer genes found in the Cancer Gene Census (GRCh38 COSMICv86) [27], a growing catalogue of mutations causally implicated in cancer, which is an enrichment greater than expected by chance (χ^2^ = 60.75 with Yates correction, *P*<0.0001; **S1 Text**). Another 64 SB candidate driver genes, including *ZMIZ1*, *ZMIZ2*, and *KMT2C*, have human orthologs with recurrent non-silent mutations identified by exome sequencing [1, 2, 28] of 68 cuSCC genomes (χ^2^ = 23.70 with Yates correction, *P*<0.0001; **S1 Text**). To investigate whether the human homologs of the mouse SB events are gained or lost in human tumors, we interrogated the limited data for CNAs in cuSCC tumors published for 40 tumors [29] which identified new and confirmed known recurrent chromosomal gains and losses in cuSCC. We found that *KDM4C* lies within the region of Chr9p frequently lost, while *NOTCH1* (Chr9q), *RASA1* (Chr5q) and *KMT2C* (Chr7q) lie within gained chromosomal regions. *KMT2C* is a known tumor suppressor in several solid tumors. Interestingly, copy-number analysis of cuSCC lymph node metastases [30] showed that *NOTCH2* (Chr1p12) and *CREBBP* (Chr16p13) lie within chromosomal regions lost, while the chromosomal region containing *TGFBR2* (Chr3q22) can be either lost or gained, and genomic regions containing *NOTCH1* (Chr9q), *TCF7L2* (Chr10q25) and *AKT3* (Chr1q) are gained. 13 genes, including PTEN and TGFBR2, two well-documented tumor suppressor genes, contain mutations in cuSCC lymph node metastases, while PTEN is also lost in cuSCC [29]. It is worth pointing out that missense and nonsense mutations in *NOTCH1* are frequent, early events in cuSCC [2], suggesting that amplified genomic regions may contain genes that are deregulated through other mechanisms. Further, *NOTCH2* is frequently mutated in cuSCC. From these data, we conclude that, while the human homologs of the SB events can be deleted in cuSCC, it appears that missense and nonsense mutations are much more frequent events. We conclude from these findings that SB identifies the same genes mutated by the diversity of mutational processes operating within end-stage human cuSCC genomes. Our insertion data implies that some of these mutations may be inactivating in human SCCs and warrant further investigation.

To gain insight into the biological functions of the candidate discovery drivers, we used a curated subset of the Enrichr analysis platform [31, 32] gene-set libraries for pathways and gene ontologies (see Methods) to perform biological pathway and process enrichment analysis from the cutaneous specimen cohorts. For this analysis, we first derived an integrated cuSCC driver list from the 60 SB|Onc3 cuSCCs sequenced by SBCapSeq with drivers defined from an additional 23 SB|Onc3 cuSCCs sequenced using Roche 454 and analyzed with our SBDriver Analysis pipeline (**S1 Text**). We also derived a composite list of cuKA drivers from two different cuKA datasets, one from cuKAs derived from SB|Onc3 mice sequenced by SBCapSeq and the second from cuKAs isolated from a previously published mouse model with K5-Cre; Pten^CKO/+^ and SB|Onc2 or SB|Onc3 mice [13, 33] sequenced using Roche 454. Using these integrated driver lists, we discovered statistically significant enrichment of recurrent SB candidate drivers in many signaling pathways and biological processes previously associated with human SCC, including EGFR, MAPK, NOTCH, and WNT-TGFβ (**S5 Fig** and **S9 Table**). Notably, we also observed that all SB|cuSCC tumor genomes have at least one defined driver involved in chromatin modification, consisting of chromatin and histone modification enzymes, as well as Hippo pathway genes. Further, the SB|cuSCC Trunk drivers encode proteins with significantly more known protein-protein interactions (PPI) than expected by chance (*P* = 8.55 × 10^−6^, STRING enrichment analysis; number of nodes: 144, number of edges: 124, expected number of edges: 82) as do the SB|cuKA trunk drivers (*P* = 6.93 × 10^−5^, STRING enrichment analysis; number of nodes: 37, number of edges: 14, expected number of edges: 4; S6 **Fig**). PPIs among drivers identified in SB-driven cuKAs suggests that perturbation of NOTCH signaling may be an initiating or acquired event in cuKA.

**Fig 4:**
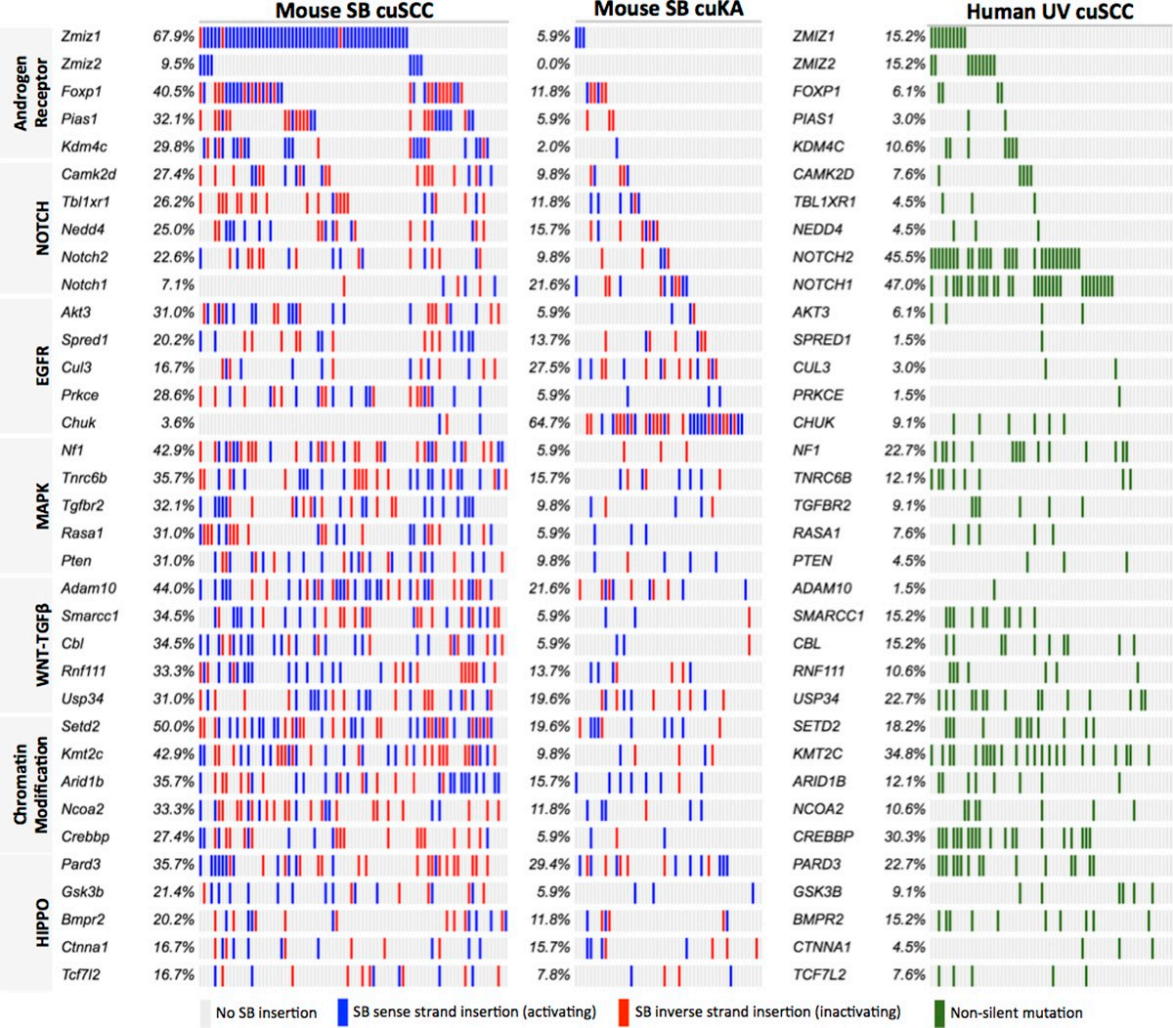
Comparative oncogenomic landscape of candidate driver mutations and common altered pathways in mouse and human cuSCC genomes. Integrated Oncoprints sorted by biological pathway or process discovery significance within the SB|cuSCC dataset and displaying the mutation burden across SB-induced mouse cuSCC (n = 84) or cuKA (n = 62) and human UV-IR-induced cuSCC genomes (**S1, S2, and S3 Data and S1 Text**).

Several of the top 30 most frequent drivers in SB cuSCC are key players in multiple pathways, suggesting that SB perturbation of multi-functional genes drives cuSCC. An oncoprint aligning the conserved drivers between SB cuSCC, SB cuKA and mutated human homologs in cuSCC that map to enriched pathways is shown in **Fig 4** Comparative genomic integration of the SB defined cuSCC and cuKA drivers with human cuSCC genomes reveals that while differences in the incidence of individual genes across cohorts can vary substantially, the biological pathways are conserved (**S5 Fig** and **S9 Table**). These differences may be due in part to the types of alterations produced by SB transposition (gene activation/inactivation) versus UV-IR (silent/non-silent mutations) or, alternatively, may reflect the complexity of the systems of genetic networks [34] that exists between driver genes and keratinocyte transformation phenotypes across species.

### Clinical relevance of SB|cuSCC driver orthologs in human SCC

The mammalian paralogous genes *Zmiz1* and *Zmiz2* are mutually exclusive trunk drivers activated in three quarters of SB-driven cuSCC genomes in an SB-driven cuSCCs, suggesting that *Zmiz* proteins are proto-oncogenes in cutaneous squamous keratinocyte transformation *in vivo*. *Zmiz1* and *Zmiz2* encode transcription factor proteins that share core C-terminal features, including androgen receptor (AR) binding domain, proline-rich binding domain, and MIZ-type zinc finger PIAS (protein inhibitor of activated STAT) domains, that coordinately act to both interact with and regulate the activity of various cancer-associated proteins [35–43]. *ZMIZ1 (RAI17/ZIMP10)* and *ZMIZ2 (ZIMP7)* are collectively mutated or amplified in one third of human cuSCC [1, 2] and 5% of hnSCC [44] genomes.

We also sought to investigate the oncogenic potential of ZMIZ1 and ZMIZ2 in SCCs using gene expression analysis from patient samples. Because there are no publicly available expression sets for cuSCC, we utilized human TCGA head and neck SCC (hnSCC) datasets, for which gene expression (RNA-seq) and patient survival outcomes are available, as a proxy for SCCs. We performed a metagene expression analysis of *ZMIZ1*, identifying a 23-gene signature that links high expression with poor outcomes in human hnSCC patients with a correlation threshold of 0.65 (Cox Proportional Hazards Regression, *P* = 0.0195; Log-rank Test, *P* = 0.028 at 50% quintile; **S7 Fig**).

From our SB-driven cuSCC model, we observed that all *Zmiz*-mutated cuSCC genomes contained one or more inactivating SB mutations in chromatin-remodeling genes (**Fig 4**), suggesting this class of mutated tumor suppressor genes may contribute significantly in driving cuSCC progression. Four of the top-ranked chromatin remodeling trunk driver orthologs were also found to be significantly mutated in >15% of human cuSCC genomes [1, 2]. Importantly, as observed in mouse tumors (**Fig 2**), mutations in the orthologs *KMT2C (MLL3)*, *ARID1B (BAF250B)*, *NCOA2 (KAT13C)*, and *CREBBP (CBP/KAT3A)* were also found in the same cuSCC genomes harboring *ZMIZ* mutations (**Fig 4**), suggesting they might cooperate during human keratinocyte transformation or tumor progression. Previously, *KMT2C* (*MLL3*) mutations were found to be associated with poor outcomes in aggressive cuSCC [1], suggesting mutations in this gene may be a biomarker for advanced stage disease.

### Gene expression alterations associated with Zmiz1 ^ΔN185^ in cuSCC genomes

SB activating trunk insertions in *Zmiz1* or *Zmiz2* are unique to SB-induced cuSCC tumors and are predicted to activate gene expression by driving expression of specific N-terminal truncated forms of Zmiz1^ΔN185^ [7] and Zmiz2^ΔN184^, a closely related paralog (**S2A and S2B Fig**). The likelihood of achieving unique, sense-strand oriented SB insertion events into two related genes at the same protein coding region on two different chromosomes in dozens of tumors from independent mice by chance is incredibly small (*P*<0.0001), suggesting both trunk driver ZMIZ oncoproteins contribute directly to keratinocyte initiation and tumor progression *in vivo*. The ZMIZ1^ΔN185^ and ZMIZ2^ΔN184^ onco-proteins share 62% sequence identity and retain both the zinc finger PIAS, proline-rich transactivation and AR binding domains. Importantly, both oncoproteins lack the N-terminus in the full-length protein, which is thought to inhibit the intrinsic transcriptional activity of a C-terminal proline-rich transactivation domain [42].

**Fig 5:**
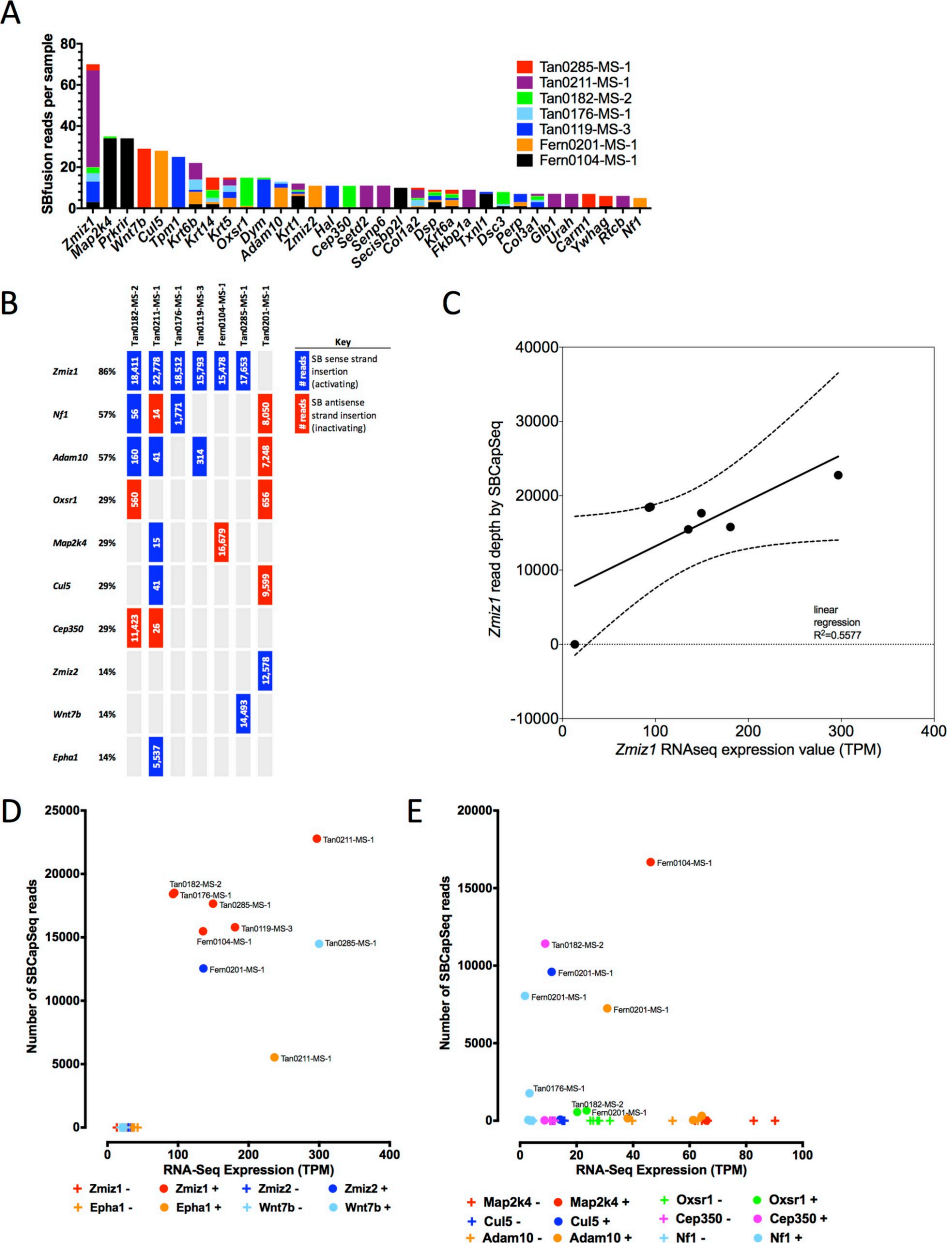
Clonally selected SB insertion events affect trunk driver gene expression in SB-induced cuSCC genomes. Seven cuSCC genomes with mutually exclusive high-read depth *Zmiz1* or *Zmiz2* SB insertions were selected for RNA-seq analysis of SB-fusion transcripts and global gene expression levels. (**A**) Genes with the most abundant SBfusion reads detected by wtRNA-seq analysis of the 7 cuSCC tumors. (**B**) Predicted gene expression effects of SB insertions in 10 drivers with high read-depth SB insertions. (**C**) High positive correlation between SBCapSeq read depth and *Zmiz1* expression by wtRNA-seq analysis. (**D**) Multifold induction of gene expression in cuSCC masses with (**+**) high read depth activating SB insertion events among 4 candidate oncogenic drivers compared with normal gene expression levels in cuSCC tumors without (–) SB insertions. Individual gene plots may be found in (**S14 and S15 Tables**). (**E**) Reduced gene expression in cuSCC masses with (**+**) high read depth inactivating SB insertion events among 6 candidate tumor suppressor drivers compared with normal gene expression levels in cuSCC tumors without (–) SB insertions. Individual gene plots may be found in (**S14 and S15 Tables**).

To analyze the expression of SB insertion-driven transcripts for *Zmiz1*, *Zmiz2* and other drivers (SBfusion reads [9]) in cuSCC genomes identified by high-insertion read depth from our genomic sequencing results, we sequenced ribo-depleted, whole-transcriptome RNA-seq (wtRNA-seq) libraries created from 7 cuSCC tumors with activating SB insertions into either *Zmiz1* (n = 6) or *Zmiz2* (n = 1) (S10 **Table**). The wtRNA-seq reads contained spliced chimeric transposon splice-donor or splice-acceptor sequences fused to adjacent known exons in the mouse genome. In total, we identified 2,906 SBfusion wtRNA-seq reads from 889 unique RefSeq genes (**S11 Table**), including 154 progression and 27 trunk cuSCC drivers, respectively (**S12 and S13 Tables**), which is substantially more than expected by chance (Chi-square test with Yates correction, χ^2^ = 159, *P*<0.0001). Thirty-three genes with SBfusion events were supported by ≥5 SBfusion reads, including our top trunk drivers *Zmiz1* and *Zmiz2* and several keratins (**Fig 5A**). We observed a positive correlation between clonal activating insertions with high read-depth SB insertions (**Fig 5B**) and high expression of SBfusion reads in *Zmiz1* (**Fig 5C**). Importantly, in each tumor, SBfusion reads containing either *SB-Zmiz1* or *SB-Zmiz2* spliced exons were represented in the top 2% of SBfusion transcripts and were supported by multiple independent SBfusion reads (**S14 and S15 Tables**). Further, *Zmiz1* or *Zmiz2* gene expression was significantly increased within each of the 7 cuSCC genomes (⪆100 and up to 300 times normal expression levels) (**Figs 5D**and **S9A, S9B, S9C, S9D, S9E, and S9F**). This strongly suggests that the SB insertion events driving either *Zmiz1* or *Zmiz2* have been clonally selected to increase oncogenic expression of the N-terminal truncated forms during keratinocyte transformation and cuSCC progression *in vivo*. We interrogated the expression of SBfusion reads from 8 additional genes in this tumor subset with high read depth insertion sites (**Fig 5C**). *Epha1* and *Wnt7b* had activating SB insertions with high SBCapSeq read depths and wtRNA-seq SBfusion reads resulting in significantly higher gene expression (⪆100 times) compared to basal expression levels observed in tumors without insertions or SBfusion reads (**Figs 5D, S8E, and S8F**). Since SB insertions in *Epha1* and *Wnt7b* were not recurrent at the population level, these genes were not identified as statistically significant drivers in cuSCC. However, the integrated genomic and transcriptomic analysis strongly suggests that each of these genes may have a ‘private’ role in driving tumor progression in the genomes in which they occur with elevated expression levels. Both genes encode proteins that are linked to cancer hallmarks—*Epha1* is an ephrin receptor subfamily of the protein-tyrosine kinase family [45] and *Wnt7b* is a WNT family member [46]. For the 6 predicted inactivated genes, *Nf1*, *Adam10*, *Oxsr1*, *Map2k4*, *Cul5*, and *Cep350* we observed reduced gene expression that correlated with high SBCapSeq read depth of inactivating SB insertions and truncated SBfusion reads (**Figs 5E,** and **S9A, S9B, S9C, S9D, S9E, and S9F).** *Adam10*, *Cul5*, and *Nf1* are Trunk Drivers and *Map2k4* is a Progression Driver in cuSCC cohorts. *Oxsr1* and *Cep350* were private selected events in individual tumors that may operate in cuSCC progression within the tumors that harbor clonal insertion events. Taken together, these data demonstrate that integrated genomic and transcriptome analyses may provide a means to discover significant cooperating drivers in cuSCC genomes from bulk specimens.

**Fig 6:**
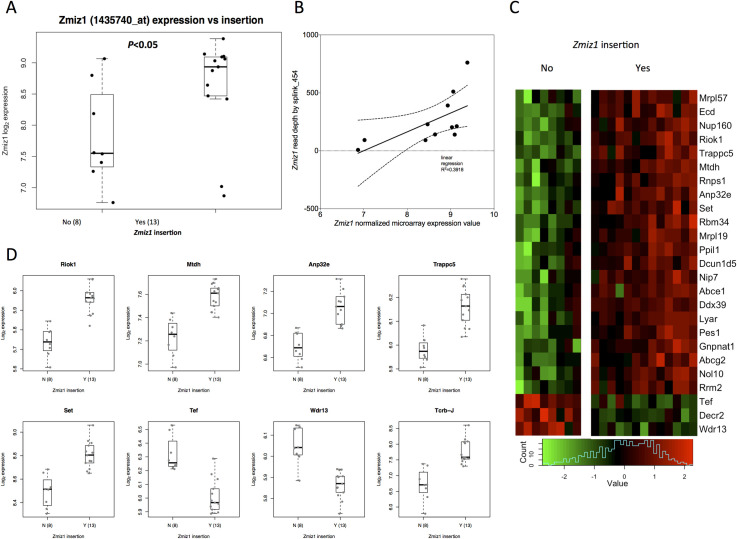
Microarray gene expression analysis revealed that tumors with *Zmiz1* SB insertions (yes) had significantly higher expression of *Zmiz1* compared to tumors without insertions (no) (**A**). Box boundaries indicate interquartile range; whiskers indicate maximum and minimum values; center lines indicate medians. (**B**) Insertion read depth for SB in the *Zmiz1* locus positively correlates with *Zmiz1* expression by microarray analysis. (**C**) Top 25 differentially expressed genes between genomes with (yes) or without (no) *Zmiz1* SB insertions (**D**) Normalized gene expression of top differentially expressed genes, *Riok1*, *Mtdh*, *Anp32e*, *Trappc5*, *Set*, *Tef*, *Wdr13*, *Tcrb-J* with corrected *P*<0.05 from **panel C**, in cuSCCs with SB-driven *Zmiz* ^**△N185**^ expression. Additional KA tumors from *Pten*-sensitized mice using keratinocyte-specific SB mutagenesis (**S1 Text**).

Next, to investigate the transcriptional signatures associated with SB-driven Zmiz1^ΔN185^ in end-stage cuSCC, we selected 13 cuSCCs with activating *Zmiz1* insertions and 9 end-stage cuSCCs lacking insertions in *Zmiz1* to perform microarray analysis. We confirmed that SB insertions drive increased expression of truncated *Zmiz1* by RT-PCR and by normalized microarray data analysis, where cuSCC tumors with *Zmiz1* insertions were found to have significantly higher mRNA abundance than tumors without *Zmiz1* insertions (**Fig 6A and S16 and S17 Tables**). We also observed a positive correlation between high-read-depth SB insertions among the 13 tumors harboring trunk *Zmiz1* insertions and increased normalized *Zmiz1* gene expression (**Fig 6B**). Differential expression analysis of the microarray data using limma from tumors with and without SB insertions in *Zmiz1* identified 432 probe sets from 355 genes significantly differentially expressed genes with FDR adjusted *P*<0.2 (**S18 Table**). A heat map of normalized expression plots for the top 25 most differentially expressed genes is shown in **Fig 6C.** Differential expression plots for the top 8 most differentially expressed genes (*P*_*FDR*_<0.05) is shown in **Fig 6D**. *Mtdh1* encodes metadherin, also known as AEG-1, a downstream target of Ras and c-Myc, and *MTHD1* is upregulated in many cancers [47]. *Set* encodes the SET nuclear proto-oncogene, which interacts with several histone modifying proteins, including CREBBP, a driver identified in our SB cuSCC tumors and KMT2A, a family member of KMT2C, also identified as a driver in our screen. Taken together, our microarray gene expression analysis demonstrates that the ZMIZ1^ΔN185^ isoform may be a critical mediator of gene expression changes essential for early keratinocyte transformation and sustained during cuSCC progression to end-stage tumors.

### ZMIZ1 ^ΔN185^ expression transforms human cutaneous keratinocytes *in vitro*

**Fig 7:**
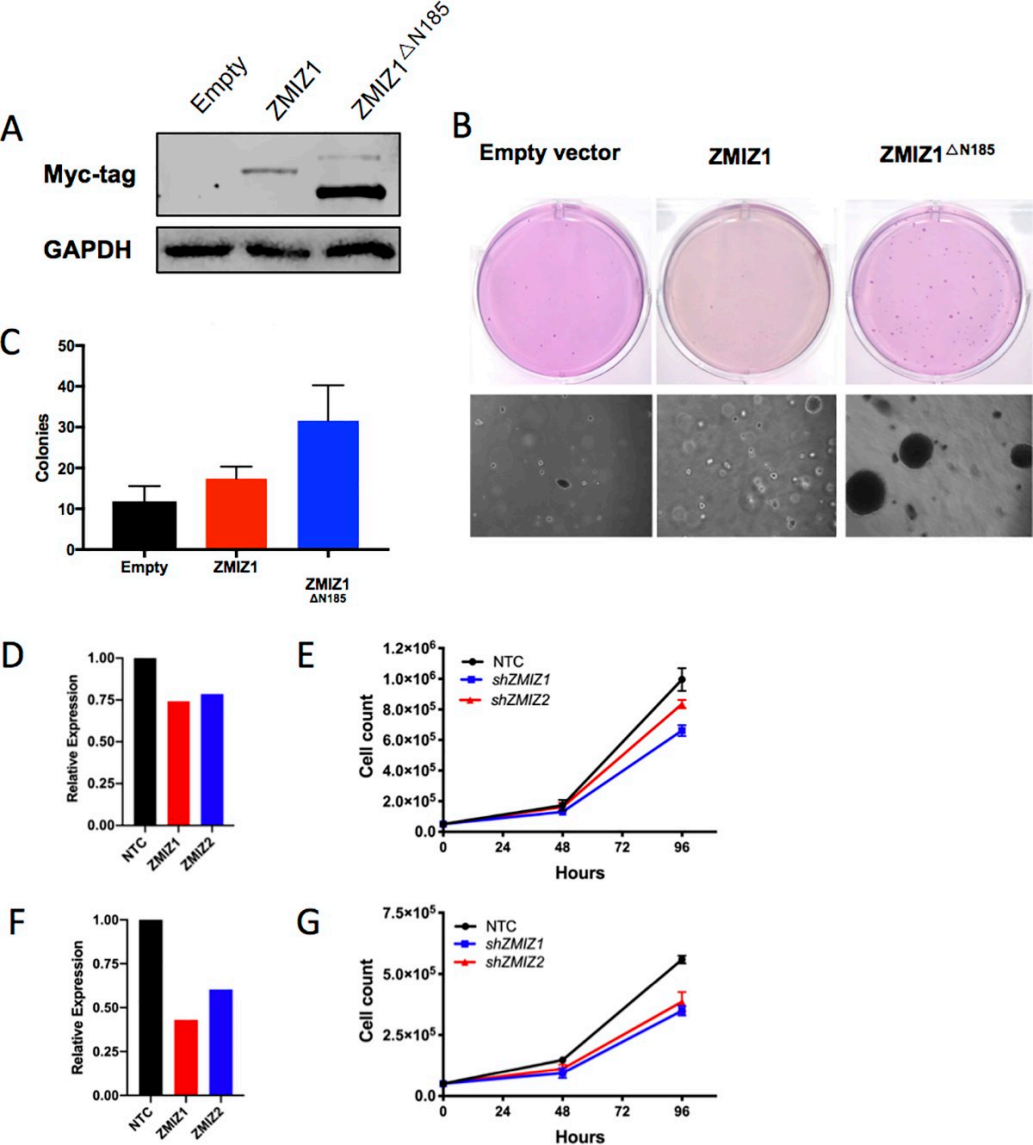
ZMIZ1^△N185^ expression transforms immortalized keratinocytes. HaCaT cells were transfected with a Myc-tagged cDNA for full length or the truncated ZMIZ1^△N185^ isoform (**A**) and protein expression was confirmed using a Myc antibody. (**B**) Colony-formation assays showed that HaCaT cells with ZMIZ1^△N185^ expression form a greater number of colonies (top inset) and larger colonies (bottom inset) relative to HaCaT cells expressing the myc-tagged full length ZMIZ1 or empty vector control. Depletion of ZMIZ1 and ZMIZ2 in cuSCC cell lines decreases cell proliferation. Stable shRNA-mediated depletion of *ZMIZ1* or *ZMIZ22* in human cuSCC cell lines (**D-E**) A431 or **(F-G)** COLO16 confirmed by TaqMan analysis relative to cells transduced with a non-targeting shRNA (NTC). *ZMIZ1* or *ZMIZ2* depletion resulted in significantly decreased cellular proliferation in (**E**) A431 cells (overall *P*<0.0001; shNTC vs. sh*ZMIZ1* q<0.0001; shNTC vs. sh*ZMIZ2 q* = 0.003; sh*ZMIZ*1 vs. shZMIZ2 *q* = 0.0023). (**F**) COLO16 (overall *P* = 0.0005; shNTC vs. sh*ZMIZ1 q*<0.0001; shNTC vs. sh*ZMIZ2 q*<0.0001; sh*ZMIZ1* vs. sh*ZMIZ2 q* = 0.18). Statistical significance measured by 2-factor ANOVA followed by multiple pairwise comparisons and FDR-adjusted.

The selection of SB insertions promoting up-regulation of a truncated *Zmiz1* transcript suggests that ZMIZ1 ^**ΔN185**^ may be an early transforming event during cuSCC development. To test this hypothesis, we first generated stable overexpression of either ZMIZ1 or the truncated ZMIZ1 ^ΔN185^ in the spontaneously immortalized human cutaneous keratinocyte HaCaT cell line [48]. Interestingly, we noted that ZMIZ1 ^ΔN185^ was more highly expressed at the protein level compared to full-length ZMIZ1, despite similar transduction efficiencies (**Fig 7A**). This observation is in line with published data by Rogers et al. 2013, where they expressed ZMIZ1 ^ΔN185^ with greater stability than full-length ZMIZ1 in various cancer cell lines. Next, we assessed anchorage-independent growth as an *in vitro* surrogate for the earliest stages of tumor development in HaCaT cells stably transduced with empty vector, full-length ZMIZ1 or ZMIZ1 ^ΔN185^. Soft agar assays showed that ZMIZ1 ^ΔN185^ expression resulted in significantly increased colony formation compared to empty vector or full-length ZMIZ1 (*P* = 0.0004, one-factor ANOVA, **Figs 7B and 7C**). These data suggest that ZMIZ1 ^ΔN185^ has a distinct function from full-length ZMIZ1 and that it promotes transformation in an oncogenic manner. Next, we investigated whether ZMIZ1 functions as an oncogene in established cuSCC. We stably knocked-down total *ZMIZ1* and showed a significant decrease in cell proliferation with *ZMIZ1* depletion compared to a controlled scrambled shRNA at 96 hours for cuSCC cell lines A431 (**Fig 7D and 7E**, **2**-factor ANOVA overall *P* = 0.0045; FDR-corrected q-values for multiple testing *q*<0.0001) and COLO16 (**Fig 7F and 7G**, 2-factor ANOVA overall *P* = 0.0005; FDR-corrected q-values for multiple testing *q*<0.0001). We showed similar results when we knocked down the *ZMIZ1* paralog *ZMIZ2* in each cell line [49]. Taken together, these data suggest that ZMIZ1 ^ΔN185^ is an oncogene that drives transformation of human cutaneous keratinocytes *in vitr*o, and that *ZMIZ1* functions as an oncogene in established cuSCCs. Further experiments are needed to address the direct role of ZMIZ1 ^ΔN185^ in promoting cuSCC initiation.

### Knockdown of chromatin modifiers drive human cutaneous keratinocyte transformation

**Fig 8:**
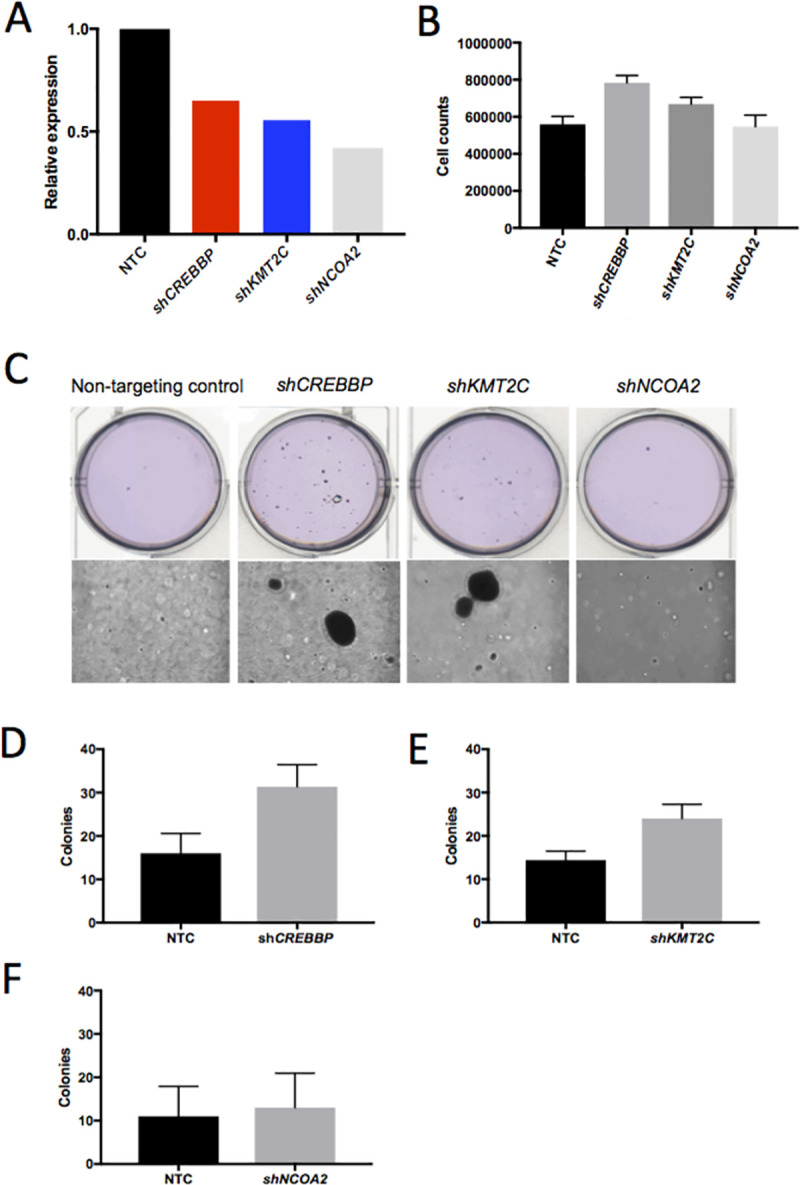
*In vitro* functional validation of chromatin remodelers in immortalized keratinocytes. Assessment of keratinocyte transformation in response to stable shRNA-mediated knockdown of *CREBBP*, *KMT2C* and *NCOA2 in* HaCaT cells (**A**) confirmed by Taq-Man showed cellular proliferation changes (**B**) with *CREBBP* knockdown relative to HaCaT cells stably transduced with a non-targeting shRNA (shNTC). Statistical significance was tested by one-way ANOVA (*P* = 0.0024) followed by Sidak’s multiple comparisons adjustment (shNTC vs. *shCREBBP*, *P =* 0.0022; shNTC vs. *shKMT2C*, *P* = 0.19; shNTC vs. *shNCOA2*, *P =* 0.99; n = 6 per group). Colony formation assays in soft agar (**C**) showed significant differences in the number and size of colonies from HaCaT cells with target gene knockdown compared to the non-targeting control for (**D**) *CREBBP* knockdown vs. shNTC, *P =* 0.018; (**E**) *KMT2C* knockdown vs. shNTC, *P* = 0.039; (**F**) *NCOA2* knockdown vs. shNTC, *P =* 0.31. Colonies were stained with crystal violet solution and quantitated; statistical significance was tested using an unpaired t-test; n = 3 per group.

Chromatin remodeling is one of the most frequently inactivated biological processes in SCC, and has been demonstrated to be involved in either tumor initiation or progression in multiple cancer types [50–55]. In our SB cuSCC screen, chromatin remodeling was the top biological process that was significantly enriched (**S5 Fig** and **S9 Table**). However, it remains unclear how expression alteration of chromatin remodeling genes function in the cuSCC disease trajectory. We prioritized three tumor suppressor genes with high concordance between SB-cuSCC and human cuSCC involved in histone modification (*KMT2C* and *CREBBP*) or transcriptional regulation (*NCOA2*) to study their roles in keratinocyte transformation using immortalized and minimally transformed HaCaT keratinocyte cell line [48]. First, we confirmed expression of each gene within HaCaT cells [49]. Next, we used shRNAs to assess whether loss of these tumor suppressors can promote cellular transformation *in vitro* as a surrogate for tumor initiation. Forced depletion of *CREBBP* significantly increased cellular proliferation relative to control (**Fig 8A, 1**-factor ANOVA, *P*<0.05) and a similar trend was observed with *KMT2C* knockdown. No significant difference in proliferation was observed between *NCOA2* knockdown and control (**Fig 8A**). To more stringently investigate transformation, we assessed anchorage-independent growth, a hallmark of cellular transformation, using a soft agar colony formation assay (**Fig 8B**). Knockdown of *CREBBP* (**Fig 8C**) or *KMT2C* (**Fig 8D**) expression resulted in larger and significantly more colonies relative to control while no significant difference was observed between *NCOA2* knockdown and control (**Fig 8E**). Taken together, these data suggest that knockdown of *CREBBP* or *KMT2C* can transform the immortalized HaCaT cell system *in vitro*.

### Knockdown of chromatin modifiers promote cuSCC tumor growth *in vivo*

**Fig 9:**
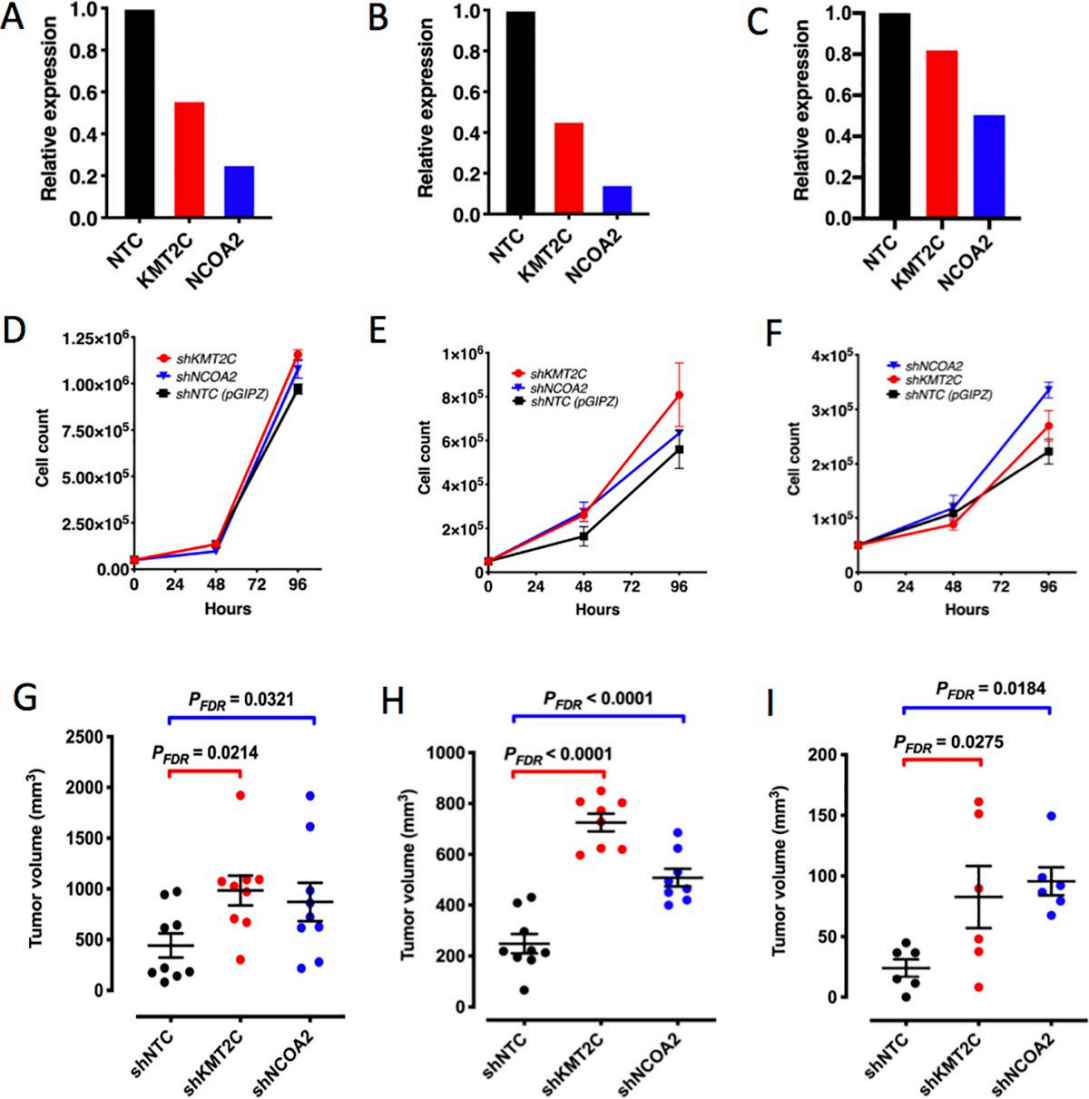
*In vitro* and *in vivo* functional validation of chromatin remodelers in cuSCC. Levels of shRNA-mediated knockdown of *KMT2C* or *NCOA2* in human cuSCC cell lines (**A**) A431 (**B**) COLO16 or (**C**) SCC13 cells confirmed by qPCR compared to a non-targeting (NTC) shRNA. Target gene knockdown significantly increases proliferation rates of (**D**) A431 (shNTC vs. sh*KMT2C q*-value<0.0001; shNTC vs. sh*NCOA2 q*-value = 0.0019; sh*KMT2C* vs. sh*NCOA2 q* = 0.017), (**E**) COLO16 (shNTC vs. sh*KMT2C q*-value = 0.01; shNTC vs. sh*NCOA2* q-value = 0.4; shKMT2C vs. shNCOA2 q = 0.06) or (**F**) SCC13 cells (shNTC vs. shKMT2C *q*-value = 0.06; shNTC vs. shNCOA2 *q*-value<0.0001; shKMT2C vs. shNCOA2 *q* = 0.01); 3 replicates per group; error bars, SEM; statistical significance measured by two-factor ANOVA followed by FDR-corrected *q-*values for multiple comparisons. *KMT2C* and *NCOA2* knockdown significantly accelerates cuSCC xenograft progression *in vivo*. (**G**) A431, corrected *P* = 0.0488 (shNTC vs. sh*KMT2C P*_*FDR*_ = 0.0214; shNTC vs. sh*NCOA2 P*_*FDR*_ = 0.0321), (**H**) COLO16, corrected *P*<0.0001 (shNTC vs. sh*KMT2C P*_*FDR*_<0.0001; shNTC vs. sh*NCOA2 P*_*FDR*_<0.0001) and (**i**) SCC13, corrected *P* = 0.0198 (shNTC vs. sh*KMT2C P*_*FDR*_<0.0275; shNTC vs. sh*NCOA2 P*_*FDR*_ = 0.0184). Statistical significance measured by one-way ANOVA followed by multiple pairwise comparisons and FDR-adjusted *P-*values.

Next, we investigated whether down-regulation of *CREBBP*, *KMT2C* or *NCOA2* could promote tumor progression in cuSCCs. First, we used shRNAs to stably knockdown these genes in established cuSCC cell lines A431, COLO16 and cuSCC13. We showed down regulation of *KMT2C* and *NCOA2* for A431 (**Fig 9A**), COLO16 (**Fig 9B**) and cuSCC13 (**Fig 9C**) by qPCR relative to cells transduced with a non-targeting control shRNA. Stable knockdown of *CREBBP* was achieved in A431 and COLO16 cells (**S10A and S10B Fig**). Next, we performed proliferation assays and demonstrated a significant increase in A431 cell proliferation with down regulation of *KMT2C* and *NCOA2* at 96 hours compared to control (**Fig 9D,** 2-factor ANOVA overall *P* = 0.0005; FDR-corrected q-values for multiple testing *q*<0.0001 for KMT2C; q = 0.0019 for *NCOA2*). Knockdown of *NCOA2* in COLO16 cells trended towards increased proliferation (**Fig 9E**) but it was not significant. However, proliferation was significantly increased with *KMT2C* knockdown at 96 hours compared to control (**Fig 9F,** FDR-corrected q-values for multiple testing *q* = 0.01). No change in proliferation was observed in A431 or COLO16 cells with *CREBBP* knockdown (**S10C and S10D Fig**). This is in contrast to the increased colony formation with *CREBBP* depletion in HaCaT cells. Finally, proliferation in SCC13 cells was significantly increased with *KMT2C* or *NCOA2* depletion compared to control (**Fig 9F,** 2-factor ANOVA overall *P* = 0.008; FDR-corrected q-values for multiple testing *q* = 0.06 for *KMT2C*; q<0.0001 for *NCOA2*). We then determined whether *CREBBP*, *KMT2C* and *NCOA2* function as cuSCC TSGs *in vivo* and performed xenograft assays by subcutaneous injection of cells for each of the three cuSCC cell lines into *NSG* immunodeficient mice. *CREBBP* depletion in A431 cells did not result in a change in tumor volume (**S10E Fig**) and this gene was not assayed *in vivo* using the other two cuSCC cell lines. All three cuSCC cell lines demonstrated enhanced *in vivo* tumor growth with *KMT2C* or *NCOA2* depletion compared to cells with a non-targeting control shRNA (**Fig 9G and 9H and 9I**, 1-factor ANOVA, *q*<0.05). Pictures of cuSCCs harvested at necropsy at defined time points of 25 days for A431 (**S11A Fig**), COLO16 at 18 days (**S11B Fig**) and SCC13 at 49 days (**S11C Fig**) show the individual tumor volumes for the control and targeted knockdown cohorts. Together, these data provide functional evidence that *KMT2C and NCOA2*, but not *CREBBP*, are human TSGs that promote *in vivo* cuSCC progression.

## Discussion

In this study, we sought to define the cooperating genetic events required for keratinocyte transformation and progression to frank cuSCC using the *SB* transposon mutagenesis system *in vivo*. Using an *Actb-Cre* transgene to activate systemic expression of the transposon, we observed tumor lesions in multiple organs. The kinetics of tumorigenesis were further accelerated in mice harboring either null (*Trp53*^*KO/+*^) [22] or recurrent point mutant (*Trp53*^*R172H/+*^) [23] alleles. This observation is consistent with previous studies showing that missense mutations in *Trp53* promote malignant transformation by abrogating both the tumor suppressive and promoting the oncogenic (gain-of-function and/or dominant negative) functions of Trp53 *in vivo* [23, 56–59]. We and others have also documented that in wild type and heterozygous sensitizing mutant mice that present with solid tumors, a much greater proportion of inactivating tumor suppressor SB insertion patterns predominate [21, 25]. While the SB|Onc3 mice developed a spectrum of tumor types, including of the hematopoietic lineage, we observed an enrichment for well-differentiated cuSCC.

Despite the high curative rate for cuSCC, there is a significant population of patients who develop advanced disease for which there are no defined therapeutic targets. Due to the high UV-induced mutation burden, combined with the scarcity of clinical samples, it is challenging to define the recurrent and druggable targets within these tumors. To address this need, we isolated early and advanced lesions from a SB mouse model of cuSCC to define recurrent drivers and potential new avenues for therapeutic intervention. From our in-depth sequencing analysis of SB insertions in advanced cuSCC genomes, we identified clonal and sub clonal drivers involved in the progression and maintenance of cuSCC. 64 and 17 drivers have human orthologs with non-silent mutations or copy number loss in human cuSCC genomes, respectively. Intriguingly, the majority of drivers in SB-driven cuSCC lesions were predicted tumor suppressor genes, suggesting that cumulative loss of these genes is a central feature of cuSCC. We showed that a few key TSGs, including *KMT2C*, are differentially expressed in cuSCC with prognostic implications for patients with hnSCC.

We subsequently focused on understanding the genetic events required for keratinocyte transformation. Our SBCapSeq and SB Driver analysis identified 349 CCDs, regardless of *Trp53* status, of which 107 have been implicated in human cancers. Intriguingly, we observed that the SB-driven cuSCC lesions were driven almost exclusively by SB inactivating insertions in tumor suppressor genes, suggesting that cumulative loss of these TSG drivers is a central feature of cuSCC. Indeed, recent analysis from our lab, using a complimentary promoterless SB transposon forward genetic screen, revealed that tumor suppressor drivers alone can lead to keratinocyte initiation and cuSCC progression *in vivo* [49]. We defined two mutually-exclusive paralogous oncogenic drivers, *Zmiz1* and *Zmiz2* among the most recurrent drivers and our report is the first to identify *Zmiz2* as a significant candidate cancer gene in any SB study and the first to discover mutual exclusivity among activated *Zmiz1*, *Zmiz2*, and *Mamld1* in early keratinocyte transformation and cuSCC progression. Insertions into *Zmiz1* [60–62] and *Mamld1* [63] have been previously observed in skin tumors induced by transposon insertional mutagenesis. The possible redundancy of ZMIZ oncoproteins in the keratinocyte transformation program, by mutual exclusivity of trunk insertions within cuSCC genomes, strongly suggests that the Zmiz1^ΔN185^ and Zmiz2^ΔN184^ oncoproteins may have common neomorphic properties that contribute to the hallmarks of skin cancer. The work of Rogers *et al*. [62] and more recently Mathios *et al*. [60] on the epigenetic regulation of *ZMIZ1* expression in human cancer, together with our finding that *ZMIZ1* and *ZMIZ2* are mutated with at least one non-silent alteration in at least one third of published human cuSCC genomes [1, 2] suggest that these genes may be an important initiating trunk mutation in human keratinocyte initiation and cuSCC progression. We confirmed this hypothesis, showing transformation of the immortalized keratinocyte HaCaT cells stably transduced with the N-terminally truncated ZMIZ1 ^ΔN185^ isoform and decreased cellular proliferation in established cuSCC lines with *ZMIZ1* or *ZMIZ2* depletion.

Intriguingly, all SB cuSCC tumors with *Zmiz1/2* insertions had inactivating insertions in at least one gene involved in chromatin remodeling, which suggests that changes in epigenetic regulation are necessary to progress a benign lesion to frank cuSCC. Our functional data suggests that *CREBBP* is involved in the earlier stages of keratinocyte transformation, while decreased *NCOA2* expression can drive disease progression. COMPASS complex members *Kmt2c* and *Kdm6a* and the switch/sucrose non-fermenting (SWI/SNF) genes *Arid1b*, *Arid1a* and *Pbrm1* were recurrently altered in our SB-cuSCC tumors. Although the COMPASS and SWI/SNF pathway members have been reported to be mutated in various cancers [64–67], whether alterations in one or cooperation of both pathways is required for the etiology of the disease remains unclear. We found that knockdown of *KMT2C*, a key molecule in the COMPASS complex, in human cuSCC cell lines accelerated *in vitro* proliferation and *in vivo* xenograft growth, supporting a role for this gene as a tumor suppressor in advanced disease. Of clinical significance, several recent studies have highlighted potential dependencies amenable to pharmacologic inhibition in cancer cells with aberrations in COMPASS members [67].

Our experimental approach for driving high mutational burdens by SB mutagenesis recapitulates the sporadic, stepwise evolutionary selection of cooperating drivers in non-melanoma skin cancers in humans exposed to solar UV. Despite these divergent mutagens, we observed remarkable overlap of driver genes, pathways, and networks between the mouse and human cuSCCs, suggesting a deep mutual biology between these shared drivers and cutaneous oncogenesis. We hope that by defining the cooperative oncogenic and tumor suppressor networks that operate during keratinocyte transformation and subsequent cuSCC progression, the results of our screen will provide a foundation for exploring new therapeutic strategies.

## Methods

### Ethics statement

Mice were bred and maintained in accordance with approved procedures granted by the respective Institutional Animal Care and Use Committees (IACUC) at the National Cancer Institute Frederick National Lab, A*STAR Biological Resource Centre, Houston Methodist Research Institute, and Moffitt Cancer Center.

### Mice used for SB screens

The following alleles were used to construct the SB-driven mouse model of multiple solid tumor histologies: *Actb-Cre* (FVB/N-Tg(ACTB-cre)2Mrt/J) [24];*Trp53*^*flox/+*^ (FVB.129P2-*Trp53*^*tm1Brn*^/Nci) [22]; *Trp53*^*LSL-R172H/+*^ (129S4-*Trp53*^*tm2Tyj*^/Nci) [23]; T2/Onc2(TG.12740) (TgTn(sb-T2/Onc3)12740Njen) [7]); and *Rosa26*-LSL SBase or SBase^LSL^; (Gt(ROSA)26Sor^tm2(sb11)Njen^) [68]). The resulting cohorts of mice were on mixed genetic backgrounds consisting of C57BL/6J, 129, C3H and FVB. Genotyping by PCR assays with primers specific to the alleles was performed. No sample size estimate was used to determine the number of mice for aging. Animals were co-housed for the duration the experiment, except in rare cases where single mice were separated based on vet recommendation due to fighting or near tumor burden end-point. Gross necropsies were performed and all masses were documented and prepared for subsequent analysis. See S1 **Fig** for details of mouse crosses. Both sexes were used for experiments. No randomization was performed; mice were assigned to groups based on genotype. No blinding was performed.

### Histological analysis

Histological analysis of spleen was performed on 5-μm sections of formalin-fixed, paraffin-embedded (FFPE) specimens stained with hematoxylin and eosin. As expected, robust nuclear staining of SB transposase (SBase) was confirmed by immunohistochemistry on FFPE tissues after antigen retrieval (pH 9) and endogenous peroxidase inhibition followed by overnight incubation with mouse antibody to SBase (anti-SBase; R&D Systems; pH 9; 1:200 dilution). After incubation with primary antibody, chromogen detection (with HRP polymer, anti-rabbit or anti-mouse, with Envision System from Dako) and hematoxylin counterstaining were performed per manufacturer’s instructions. Genomic DNA (gDNA) was isolated from flash frozen necropsy specimens using Qiagen Gentra Puregene DNA isolation kit protocol for tissue.

### Mapping transposon insertion sites using the splink_454 method

SB insertion reads were generated by 454 GS Titanium sequencing (Roche) of pooled splinkerette PCR reactions with nested, barcoded primers was performed [10, 11, 69]. Pre- and post-processing of 454 reads to assign sample DNA barcodes, filter out local hopping events from donor chromosomes, and map and orient the SB insertion sites across the entire nuclear genome of the mouse was performed. All SB insertions from donor chromosomes were filtered out prior to identification of common insertion sites using the Gaussian kernel convolution (GKC) [69, 70] and SB driver analysis [21] methods.

### Mapping transposon insertion sites using the SBCapSeq method

Full details for the SBCapSeq protocol (9) optimized for sequencing from solid tumors, will be published elsewhere (Mann *et al*., in review; for general protocol and concept, see [9, 71, 72]). Briefly, for selective SB insertion site sequencing by liquid hybridization capture, gDNA (0.5 μg per sample) of either bulk tumor specimens or single cell WGA genomes was used for library construction using the AB Library Builder System, including random fragmentation and ligation of barcoded Ion Xpress sequencing adapters. Adapter-ligated templates were purified by Agencourt AMPure beads and fragments with insert size of 200±30bp were excised, purified by Agencourt AMPure beads, amplified by 8 cycles of adapter-ligation-mediated polymerase chain reaction (aLM-PCR), and purified by Agencourt AMPure beads with elution in 50 μl of TE (1X Tris-EDTA [Ethylenediaminetetraacetic acid], pH8). Capture hybridization of single or multiplexed up to 12 barcoded libraries (60 ng per sample) was performed using custom xGen Lockdown Probes (IDT; full details available at https://doi.org/10.35092/yhjc.11441001.v1 [71]). All 120-mer capture and blocking oligonucleotide probes were purchased from IDT as Ultramer DNA Oligos with Standard Desalting. Bar-coded single and multiplex captured library fragments were further amplified by 12 cycles of LM-PCR and run using an Agilent 2100 Bioanalyzer or TapeStation to estimate enrichment. Bar-coded single and multiplex captured libraries were quantified by Qubit Fluorometer and quantitative Real Time-PCR (qRT-PCR) were used to dilute libraries for template preparation and Ion Sphere Particle (ISP) loading using Ion Chef System and sequencing on the Ion Proton platform with PI_v3_ semiconductor wafer chips per manufacturers recommended instructions. High-throughput sequencing of up to 39 multiplex captured libraries was carried out per PI_v3_ chip to achieve at least 1.5 million reads per barcode. Reads containing the transposon IRDR element were processed using the SBCapSeq bioinformatic workflow as described [9].

### SB driver analysis

BED formatted files containing SB insertions from each of the histologically verified SB-cuSCC, SB-cuKA, and SB-cuSK cohort specimens (**S1 and S2 and S3 Data**) were used to perform SB Driver Analysis to identify statistically significant discovery, progression, and trunk driver genes that contain more SB insertions than expected by chance and were recurrently altered in three or more tumors [21]. Discovery and progression SB Driver Analysis considered all SB insertion events; trunk SB Driver Analysis considered only insertions represented by 5 or more reads. Statistically significant discovery drivers were defined as adjusted *p*-values using false discovery rate (FDR) multiple testing correction (*q*-value<0.5). Statistically significant progression and trunk drivers were defined as adjusted *p*-values using family-wise error rate (FWER) multiple testing correction (FWER adjusted-*P*<0.05). Statistically significant drivers on donor and non-donor chromosomes analyses were performed separately and combined into a single driver lists (**S1 and S2 and S3 Data**). Due to a local-hopping phenomenon known to occur with SB, where transposition events are biased to occur in *cis* along the donor chromosome more frequently than in trans to non-donor chromosomes in the genome, insertions from the donor chromosome are typically filtered away computationally before candidate cancer genes are identified. However, doing so in cohorts derived from mice with only a single SB donor allele (like our cuSCC cohorts) means that genome-wide driver analysis is not possible, because all genes on the donor chromosome are censored and are not reported even though they may contribute to the overall tumor burden. Since we obtained quantitative SBCapSeq datasets in this study, we evaluated whether all genes on the donor chromosome should be censored or if perhaps, as observed in *PiggyBac* transposon genomes, only a relatively small portion of the donor chromosome locus, occurring in close proximity to the donor concatemer insertion site, demonstrate significant bias compared with the frequency of SB insertions into non-donor chromosomes. In the cuSCC cohorts sequenced with SBCapSeq, we found that donor chromosome SB insertion site frequencies are significantly higher between chr9:79,000,000 and chr9:95,000,000 and drop to the genome wide average for non-donor chromosomes over the rest of chromosome 9 (**S12 Fig**), suggesting that filtering all SB donor chromosome insertion events may not be warranted. To confirm and extend this observation, we used all tumors within the SBCDDB with chromosome 9 donor alleles and identified that the same region between chr9:79,000,000 and chr9:95,000,000 had a higher frequency of SB insertions than the rest of chromosome 9 loci, which again matched non-donor chromosome insertion frequencies (**S12 Fig**). Thus, we ran SB Driver Analysis on tumors derived from mouse cohorts harboring an SB T2/Onc3 TG.12740 donor allele chromosome 9 (cuSCC83_454, cuSCC60_SBC, and cuKA11_SBC), by excluding the 81 genes that map between *Filip1* at chr9:79663368–79825689 and *Tfdp2* at 96096693–96224065 from the driver gene output files. To define trunk drivers, we used a normalized number as a percent of the total SB insertions, 300 reads is the cut off for the top 5% of reads, based on the assumption that the distribution of SB insertions from normal skin represented a normal distribution of background events not exceeding 5% of reads. We selected a driver cut-off as a whole number that was within the 5% of the highest insertion sites from the normal skin samples recognizing that a random number of insertions may have higher read depths from technical (e.g., amplicon amplification bias or known limitations of the Ion Torrent sequencing and alignment platform of homopolymer regions) and/or biological (e.g., clonal keratinocyte distributions within the sampled histologically normal skin or insertion events linked to loci overrepresented within the sampled histologically normal skin genomic DNA) sources of variation that may permit insertion overabundance compared with the majority (~95%) of reads within the range of sequencing depths represented within the specimen pools. In sequencing the histologically distinct regions from cuSCC masses, we used a normalized number as a percent of the total SB insertions, 200 reads representing read depths in the top 30%, so as to provide more power to detect recurrent gene insertions, given that our sequencing depth was lower for this experiment. Despite lowering this threshold, we note that *Zmiz1* was the only gene that consistently had activating transposon insertions in all four independent lesions sequenced, suggesting that *Zmiz1* proto-oncogenic activation is selected early and maintained throughout clonal evolution of these histologically heterogeneous masses.

### Microarray gene expression analysis

Gene expression profiling of histologically confirmed SB-cuSCC masses selected by presence or absence of *Zmiz1* insertion by 454Splink sequencing was performed using Affymetrix microarrays, as described previously [9]. Briefly, 100ng of total RNA for each sample was extracted using a NORGEN Biotek Animal Tissue RNA Purification kit (Cat #25700) followed by labeled with an Affymetrix 3’ IVT Express kit (Cat # 901229) using the manufacturer’s instructions. Labeled samples were hybridized to Affymetrix GeneChip Mouse Genome 430 2.0 Arrays, and scanned at the University of Otago Genomics & Bioinformatics Facility. Raw data processing used R (version 3.6.1) [73] with the “rma” function of the “affy” package [74], including quantile normalization but no background correction. Quality assessment of the microarray data was performed in R using the “affyQCReport” package [75]. Differential expression was performed using limma [76], and gene set analysis of Reactome pathways was performed using ReactomePA [77]. All additional data analysis and visualization were performed using Python and R.

### Transcriptome sequencing

Total RNA was isolated from flash frozen necropsy specimens using *mir*Vana miRNA Isolation Kit (Ambion by Life Technologies, AM1560) from a shaved portion of each pathology verified cuSCC mass from 7 tumors for which sufficient tissues were available and the presence of a high read depth *Zmiz1* or *Zmiz2* trunk driver insertion was identified by SBCapSeq. Whole transcriptome RNA-Seq (wtRNA-Seq) libraries were prepared from total RNA (5 μg per sample) plus ERCC RNA Spike-In (Ambion by Life Technologies, 4456739) followed by selective ribosomal RNA (rRNA) depletion using the RiboMinus Eukaryote System v2 (Ambion by Life Technologies, A15026). rRNA-depleted total RNA (500 ng per sample) was used for whole transcriptome library construction according to the Ion Total RNA-Seq Kit for the AB Library Builder System (Life Technologies, 4482416) protocol for barcoded libraries, Ion Sphere Particle (ISP) loading using Ion Chef System and sequencing on the Ion Proton platform with PI_v3_ semiconductor wafer chips per manufacturers recommended instructions. Up to 4 RNA-Seq libraries were multiplex sequenced on a PI_v3_ chip twice to achieve >3 GB (~40 million reads) per specimen. The Life Technologies Torrent Suite software was used to perform checking of the raw sequence data before the generation of sequencing read files in FASTQ format and minimally processed mRNA-Seq and wtRNA-Seq reads were processed using the Bowtie2 [78] and Tophat [79] algorithms to align reads to a custom version of the mouse mm9+pT2/Onc genome, as described [9]. The detection of SB fusion events, defining novel SB fusion transcripts, and measuring transcript abundance in SB cuSCC tumor specimens from RNA-Seq data were performed as described [9].

### Biological pathway and process enrichment analysis

Enrichr [31, 32], an online analysis tool for human and mouse gene-set enrichment, was used to identify specific signaling pathways and processes enrichment using the various cohort candidate cancer driver genes and/or their human orthologs (**S9 Table and S1 Text**). The online STRING network enrichment analysis tool [80] was also used to determine functional connections between trunk drivers from the cuSCC and cuKA cohorts.

### Human cancer cell lines

The cutaneous squamous cell carcinoma A431(CRL–1555) and grade IV lung squamous cell carcinoma SW900 (HTB–59) cell lines, were purchased from American Type Culture Collection (ATCC) and grown according to the manufacturer’s suggested conditions in complete medium (1× DMEM (ATCC, 30–2002) for A431cells or 1× ATCC-formulated Leibovitz’s L–15 Medium, Catalog No. 30–2008 medium (ATCC, 30–2001) for SW900 cells) supplemented with 10% FBS and 1× penicillin-streptomycin grown at 37°C in 5% CO2. The cutaneous squamous cell carcinoma SCC13 cell line [81, 82], were obtained from Harvard Skin Disease Research Center (HSDRC) and grown using the human keratinocyte culture methods provided by the HSDRC. The cutaneous squamous cell carcinoma COLO16 cell line [83] were grown in DMEM/Ham’s F12 50/50 media supplemented with cholera toxin (Sigma, C0852), insulin (Sigma, I5500-50MG), epidermal growth factor (Serotec/Bio-Rad, EGF-1), hydrocortisone (Sigma, H4881-1G), liothyronine (Sigma, T6397-100MG) and apo-transferrin (Sigma, T2252-100MG). All cell lines were verified human origin and free from pathogens and mycoplasma. Mycoplasma infection monitoring was performed using MycoAlert Detection Kit (Lonza) and only mycoplasma-free cultures were used.

### Generation of stable shRNA-expressing cell lines

High titer lentiviral particles for pGIPZ-shRNA constructs targeting each of the 12 cutaneous candidate driver genes, and one non-targeting control, were purchased from Thermo Scientific Open Biosystems (**S19 Table**). Cells were plated at a density of 5 × 10^4^ cells per well in a 24-well plate in complete media 24 hours prior to infection. The following day, cells were plated with serum-free culture medium containing 8 μg/mL polybrene (Millipore), and transduced as pools of three independent shRNAs to different exons of each target gene, or individually for the control, at multiplicity of infection of 6. Puromycin selection was added the following day at concentrations of 1.0 to 3.0 μg/mL puromycin (Thermo Fisher Scientific) in complete media, and replaced every three until stable lines were achieved.

### Proliferation assay

To assess cuSCC progression, cuSCC cell lines were seeded in 6 well plates at a density of 5 × 10^4^ cells per well in complete media. Cell numbers for each condition were counted after 48- and 96-hour post-seeding. To assess transformation by proliferation, HaCaT cells were seeded at a density of 5 × 10^3^ cells per well and cell counts performed after seven days.

### Soft agar assay

To assess anchorage-independent growth, HaCaT cells were seeded at a density of 1 × 10^4^ cells per well in a six well plate in 0.3% agar, over a layer 0.6% agar. Complete media was added to each well the following day, and replaced every two days for four weeks. At end point, cells were fixed in 20% methanol and 0.0025% crystal violet. Washes were done with milliQ water until low background was achieved, and plates were scanned, and colonies counted for each condition. Zmiz1 full length, Zmiz1^FL^ (pLV[Exp]-EF1A>{mZmiz1[NM_183208.4](ns)*/Myc/FLAG:P2A:TurboRFP(ns):P2A:Puro), N-terminally truncated, Zmiz1^ΔN185^ (pLV[Exp]-EF1A>{mZmiz1[deltaN185 -NM_183208.4]*(ns)}/Myc/FLAG:P2A:TurboRFP(ns):P2A:Puro), or empty vector control, EV (pLV[Exp]-EF1A>TurboRFP(ns):P2A:Puro), expression vectors were custom synthesized using the VectorBuilder platform (Cyagen). Cells were transfected with the expression plasmids and selected with 800ug/mL G418 to obtain stable cell lines.

### qRT-PCR

Total RNA was purified and DNase treated using the RNeasy Mini Kit (Qiagen). Synthesis of cDNA was performed using SuperScript VILO Master Mix (Life Technologies). Quantitative PCR analysis was performed on the QuantStudio 12K Flex System (Life Technologies) or 7900HT Sequence Detection System (Applied Biosystem). All signals were normalized to the levels of GAPDH TaqMan probes. TaqMan probes were obtained from Life Technologies (**S20 Table**).

### Xenografts

One million cells were prepared for injection into the left flank of randomized selected male and female immunodeficient NSG (NOD.Cg-Prkdcscid;Il2rgtm1Wjl/SzJ; JAX, 005557; 6–15 weeks old) mice. Allocation to study groups was random. Xenograft measurements were taken twice weekly using digital calipers while mice were conscious but restrained by an experimenter familiar with collecting caliper measurements of xenografts and blinded to the experimental group designations. GFP fluorescence was visualized at the time of caliper measurement. Ellipsoid tumor volumes were calculated as volume (mm3) = 0.52(length (mm) × width2 (mm2)), where the two longest axes, length and width, were the major and minor diameter measurements, respectively; width2 represents an assumption that the xenograft depth was equivalent to the diameter of the minor axis. Statistical significance of tumor volumes at defined time points was determined by either one-way t test for all cohorts relative to shNTC or by two-way, repeated-measures ANOVA with Bonferroni correction for multiple comparisons, as indicated.

### Western blot

Whole cell or tissue lysates were prepared in RIPA buffer supplemented with protease and phosphatase inhibitors, and samples sonicated to achieve optimum lysis. Protein concentrations were quantitated using the BCA assay, and 20 μg of lysates were loaded into 8% SDS-polyacrylamide gels. Gels were resolved at 80V for 120 mins, transferred onto nitrocellulose membrane for 70 mins at 0.35A with transfer buffer containing 20% methanol, and blocked for one hour at room temperature with 5% w/v BSA in 0.1% TBST. Membranes were incubated with primary antibodies in 5% w/v BSA in 0.1% TBST. Antibodies used in this study are: Total ERK (1:1000, Cell Signaling #9102), phospho ERK (1:1000, Cell Signaling #4695), GAPDH (1:5000, Santa Cruz sc-69778), anti-mouse and anti-goat secondary antibodies (1:10000, LiCOR IRDye 680/800 Cat. 925-68070/926-32211). Blots were imaged using the LiCOR Odyssey Fc system.

### Software

Unless otherwise noted, bioinformatic analysis pipelines, report generation, and figure visualization were performed using bash, R (version 3.6.1), Python scripts, and GraphPad Prism 8 software (Version 8.1.1). Hierarchical clustering was performed in Python 2.7.10 with the scipy 0.13.0b1 toolbox using the Hamming distance metric with Ward’s linkage method. The R packages ggplot2 [84] and Gviz [85] were used to generate the graphics in various figure panels.

### URLs

TgTn(sb-T2/Onc3)12740Njen; Gt(ROSA)26Sortm2(sb11)Njen double mutant mice, https://ncifrederick.cancer.gov/Lasp/MouseRepository/MouseModels/StrainDetails.aspx?StrainNum=01XGB&g=ROSA26; FVB/N-Tg(ACTB-cre)2Mrt/J mice, http://jaxmice.jax.org/strain/003376.html; Cancer Gene Census, http://cancer.sanger.ac.uk/census/; Mouse Genome Informatics (MGI) database, ftp://ftp.informatics.jax.org/pub/reports/index.html; Enrichr, http://amp.pharm.mssm.edu/Enrichr/, STRING, https://string-db.org/, The Cancer Genome Atlas (TCGA), http://cancergenome.nih.gov/; FVB.129P2-Trp53tm1Brn/Nci mice, https://ncifrederick.cancer.gov/Lasp/MouseRepository/MouseModels/StrainDetails.aspx?StrainNum=01XC2&g=Trp53; 129S4-Trp53tm2Tyj/Nci mice, https://ncifrederick.cancer.gov/Lasp/MouseRepository/MouseModels/StrainDetails.aspx?StrainNum=01XM2&g=Trp53; immunodeficient NSG (NOD.Cg-Prkdcscid;Il2rgtm1Wjl/SzJ) mice, https://www.jax.org/strain/005557.

### Accession codes

NCBI BioProject accessions: PRJNA580460 for whole-transcriptome RNA-Seq data; PRJNA580462 for microarray expression data.

## Supporting information

S1 Fig Overview of genetic crosses to generate SB|Trp53|Onc3 mouse model. Schematic of genetic crosses to generate the genetic cohorts aged for tumor development in this study.
(TIFF)

S2 Fig SB insertion patterns in activated and inactivated drivers. Recurrent TA-dinucleotide SB insertion events from individual cuSCC genomes reveal activating sense strand SB insertions in the locus of the paralogs *Zmiz1* (**A**) or *Zmiz2* (**B**). Chimeric fusion between the SB transposon splice donor (SD) and splice acceptor sites at exon 9 of *Zmiz1* or exon 6 of *Zmiz2* results in CAG-promoter driven transcription of a truncated mRNA predicted to encode N-terminally truncated *Zmiz1*^ΔN-185 aa^ or *Zmiz2*^ΔN-184 aa^ containing functional MIZ-type zinc finger domains and a Siz/PIAS RING finger (SP-RING). Representative SB insertion maps in cuSCC trunk drivers showing the locations of mapped SB insertions (triangles) predicted to inactivate tumor suppressors, shown in detail for *Pten* (**C**). Inactivating insertion maps for chromatin remodelers *Ncoa2* (**D**) and *Kmt2c* (**E**).
(TIFF)

S3 Fig Evaluating the reproducibility of SBCapSeq results from bulk cuSCC and normal skin specimens. Representative plots of individual SB insertion sites based on read depth from two individual cuSCC genomes (**A-B**) and two unselected skin cell genomes from histologically normal skin (**C-D**) using genomic DNAs isolated from bulk tumor specimens from biological replicate libraries (independent library workflows applied to the same biological specimen isolate) comparing library 1 (*x*-axis) to library 2 (*y*-axis). Biological reproducibility of cuSCC specimen libraries is indicated by both Pearson’s and Spearman’s correlation metrics (**A-B**). No biological reproducibility of normal skin specimen libraries is observed (**C-D**) indicated by negative values for Pearson’s and Spearman’s correlation metrics and a failure to identify the same SB insertion sites. Individual SB insertion sites plotted by read depth from two technical replicates (the same library preparation) from two separate sequencing runs (**E-F**) confirms high reproducibility between individual libraries at high-to-moderate read depths, supported by both Pearson’s (r = 0.48) and Spearman’s (ρ = 0.49 or undetermined) correlation metrics.
(TIFF)

S4 Fig Hierarchical two-dimensional clustering of recurrent events in cuKA and cuSCC. Hierarchical two-dimensional clustering (Hamming distance with the Ward method of agglomeration) of recurrent genic SBCapSeq insertion events from four skin masses containing distinct cuKA and cuSCC regions. Four distinct specimen (*x*-axis) and gene (*y*-axis) clades, each pertaining to a single bi-lesional mass, demonstrate clonal identities. *Zmiz1* was the only gene recurrently mutated across all samples with high read depths, highlighted in blue text.
(TIFF)

S5 Fig Curated biological pathways and processes enriched within SB-induced cuSCC. Table of significant pathways collated from pathway enrichment categories.
(TIFF)

S6 Fig Trunk driver STRING network analysis. (**A**) The SB|cuSCC Trunk Driver (n = 84) network has significantly more known protein-protein interactions than expected by chance (*P* = 8.55 × 10^−6^8.55e-06, STRING enrichment analysis; number of nodes: 144, number of edges: 124, expected number of edges: 82 (**B**) The SB|cuKA Trunk Driver (n = 62) network also has significantly more known protein-protein interactions than expected by chance (*P* = 6.93 × 10^−5^, STRING enrichment analysis; number of nodes: 37, number of edges: 14, expected number of edges: 4.
(TIFF)

S7 Fig *ZMIZ1* metagene within the TCGA Head & Neck Squamous Cell Carcinoma (hnSCC) RNA-seq dataset. (**A**) *ZMIZ1* metagene heatmap constructed using singular value decomposition of human hnSCC RNA-seq dataset from TCGA consisting of a 23-gene signature. Multivariate analysis of a gene signatures that correlates with the expression of *ZMIZ1* in HNSCC. (**B**) Survival plots for patients with head and neck type SCC (hnSCC) based on the *ZMIZ1*–centric metagene.
(TIFF)

S8 Fig Clonally selected SB insertions affect trunk driver proto-oncogene expression in SB-cuSCC genomes. (**A**) Summary of *Zmiz1* and *Zmiz2* transcripts identified from bulk analysis of cuSCC cells by wtRNA-seq are represented as the log_10_ ratio of fragments per kilobase of transcript per million (FPKM) from the average of 6 genomes with SB insertions into *Zmiz1* compared to the transcripts from the single genome with *Zmiz2* SB insertion, defined by FPKM mapped reads from ribo-depleted RNA. (**B**) Transcripts identified from bulk analysis of cuSCC cells by wtRNA-seq are represented as the log_10_ ratio of SB insertion containing compared to wild-type transcripts, defined by fragments per kilobase of transcript per million mapped reads from ribo-depleted. (**C-F**) Individual panels from **Fig 5E**: multifold induction of gene expression in cuSCC masses with (**+**) high read depth activating SB insertion events among 4 candidate oncogenic drivers compared with normal gene expression levels in cuSCC tumors without (–) SB insertions.
(TIFF)

S9 Fig Clonally selected SB insertions affect trunk driver genes by inactivating expression in SB-cuSCC genomes. (**A-F**) Individual panels from **Fig 5F**: reduced gene expression in cuSCC masses with (**+**) high read depth inactivating SB insertion events among 6 candidate tumor suppressor drivers compared with normal gene expression levels in cuSCC tumors without (–) SB insertions.
(TIFF)

S10 Fig *CREBBP* knockdown does not alter proliferation rate in cuSCC cell lines. (**A**) 96-hour proliferation assay (n = 3, error bars SEM). (**B**) *In vivo* xenograft assay of COLO16 shNTC or shCREBBP into NSG immunodeficient mice (n = 9 per condition).
(TIFF)

S11 Fig Gross photographs of cuSCC xenograft masses collected at necropsy showing robust TurboGFP expression. Xenografts of cuSCC human cell lines A431, COLO16 and SCC13 with stable lentiviral constructs expressing shRNAs directed knockdown of target drivers *KMT2C* (shKMT2C) or *NCOA2* (shNCOA2) relative to a non-targeting control (shNTC) grown in *NSG* immunodeficient mice.
(PDF)

S12 Fig SB T2/Onc3 TG.12740 allele donor position mapping and exclusion for SB Driver Analysis. Cumulative SB insertion sites at TA-dinucleotides from (**A**) 454_Splink and (**B**) Ion_SBCapSeq datasets showing the likely TG.12740 donor site at 87,000,000 bp and the exclusion region (yellow box) between 79,000,000 and 95,000,000 bp applied when SB Driver Analysis was run on chromosome 9. The 81 genes between *Filip1* at chr9:79663368–79825689 and *Tfdp2* at chr9:96096693–96224065 were excluded from SB Driver Analysis. The complete list of 81 genes excluded from SB Driver Analysis from tumors harboring the SB T2/Onc3 TG.12740 donor allele: *1190002N15Rik*, *1700034K08Rik*, *1700057G04Rik*, *1700065D16Rik*, *4930524O08Rik*, *4930554C24Rik*, *4933400C23Rik*, *9330159M07Rik*, *9430037G07Rik*, *A330041J22Rik*, *Adamts7*, *AF529169*, *Ankrd34c*, *Atr*, *B430319G15Rik*, *Bckdhb*, *Bcl2a1a*, *Bcl2a1b*, *Bcl2a1d*, *Cep162*, *Chst2*, *Ctsh*, *Cyb5r4*, *D430036J16Rik*, *Dopey1*, *Elovl4*, *Fam46a*, *Gk5*, *Hmgn3*, *Htr1b*, *Ibtk*, *Impg1*, *Irak1bp1*, *Lca5*, *Me1*, *Mei4*, *Mir184*, *Mir6386*, *Mir7656*, *Morf4l1*, *Mrap2*, *Mthfs*, *Mthfsl*, *Myo6*, *Nt5e*, *Paqr9*, *Pcolce2*, *Pgm3*, *Phip*, *Plod2*, *Pls1*, *Plscr1*, *Plscr2*, *Plscr4*, *Plscr5*, *Prss35*, *Rasgrf1*, *Ripply2*, *Rwdd2a*, *Senp6*, *Sh3bgrl2*, *Slc9a9*, *Snap91*, *Snhg5*, *Snx14*, *Syncrip*, *Tbc1d2b*, *Tbx18*, *Tmed3*, *Tpbg*, *Trim43a*, *Trim43b*, *Trim43c*, *Trpc1*, *Ttk*, *U2surp*, *Ube2cbp*, *Xrn1*, *Zfp949*, *Zic1*, *Zic4*.
(TIFF)

S1 Table Tumor incidence and subgroup classifications by cohort. (XLSX)

S2 Table Specimen metafile data for projects sequenced using SBCapSeq protocol with Ion Torrent Proton sequencer. (XLSX)

S3 Table Discovery and progression SB Driver Analysis for cuSCC60_SBC. (XLSX)

S4 Table Trunk SB Driver Analysis for cuSCC60_SBC. (XLSX)

S5 Table Discovery and progression SB Driver Analysis for cuKA11_SBC. (XLSX)

S6 Table Trunk SB Driver Analysis for cuKA11_SBC. (XLSX)

S7 Table Discovery and progression SB Driver Analysis for cuSK32_SBC. (XLSX)

S8 Table SBCapSeq read depth and analysis for 4 cuSCC genomes selected for multi-region resequencing because they had intermixing of cuSCC and cuKA histologies. (XLSX)

S9 Table Enrichr gene set pathway enrichment analysis of cuSCC drivers. (XLSX)

S10 Table Summary of 7 cuSCC transcriptomes selected for whole transcriptome RNAseq analysis. (XLSX)

S11 Table BED file of SBfusion insertions in 7 cuSCC genomes by whole transcriptome RNAseq analysis. (XLSX)

S12 Table Venn diagram for overlap of genes with SBfusion reads detected by whole transcriptome RNAseq analysis and cuSCC60_SBC discovery driver. (XLSX)

S13 Table Venn diagram for overlap of genes with SBfusion reads detected by whole transcriptome RNAseq analysis and all cuSCC drivers. (XLSX)

S14 Table Transcripts per million (TPM) normalized whole transcriptome RNAseq values per gene from RNA isolated from cuSCC genomes with and without Zmiz1 insertions. (XLSX)

S15 Table. Fragments Per Kilobase of Transcripts per Million (FPKM) normalized whole transcriptome RNAseq values per gene transcript from RNA isolated from cuSCC genomes with and without Zmiz1 insertions (XLSX)

S16 Table Normalized microarray values per gene from RNA isolated from cuSCC genomes with and without Zmiz1 insertions. (XLSX)

S17 Table Normalized microarray values per probe from RNA isolated from cuSCC genomes with and without Zmiz1 insertions. (XLSX)

S18 Table All 289 genes with differential expression analysis from microarray data from RNA isolated from cuSCC genomes with and without Zmiz1 insertions with P<0.0001 and q<0.05. (XLSX)

S19 Table Lentiviral vectors containing shRNAs used in this study. (XLSX)

S20 Table TaqMan probes used in this study. (XLSX)

S1 Text Oncogenomic comparisons between SB candidate Trunk driver genes and their direct orthologs in human Cancer Gene Census. (DOCX)

S1 Data BED file of SB insertions for cuSCC60_SBC. (XLSX)

S2 Data BED file of SB insertions for cuKA11_SBC. (XLSX)

S3 Data BED file of SB insertions for cuSK32_SBC. (XLSX)

S4 Data BED file of SB insertions for 4 cuSCC genomes selected for multi-region resequencing because they had intermixing of cuSCC and cuKA histologies. (XLSX)

S5 Data Numerical data for graphs. (XLSX)
