# Supplementary figures and images for: Transposon mutagenesis identifies cooperating genetic drivers during keratinocyte transformation and cutaneous squamous cell carcinoma progression
Source: PLoS Genet. 2021 Aug 16;17(8):e1009094. doi: 10.1371/journal.pgen.1009094 (PMC8389471; doi:10.1371/journal.pgen.1009094)

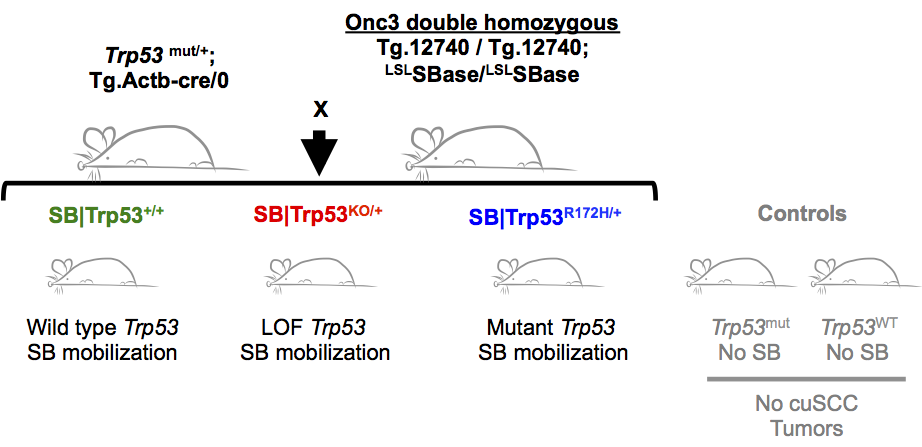

Supplement: S1 Fig — Schematic of genetic crosses to generate the genetic cohorts aged for tumor development in this study. (TIFF) [file pgen.1009094.s001.tiff]

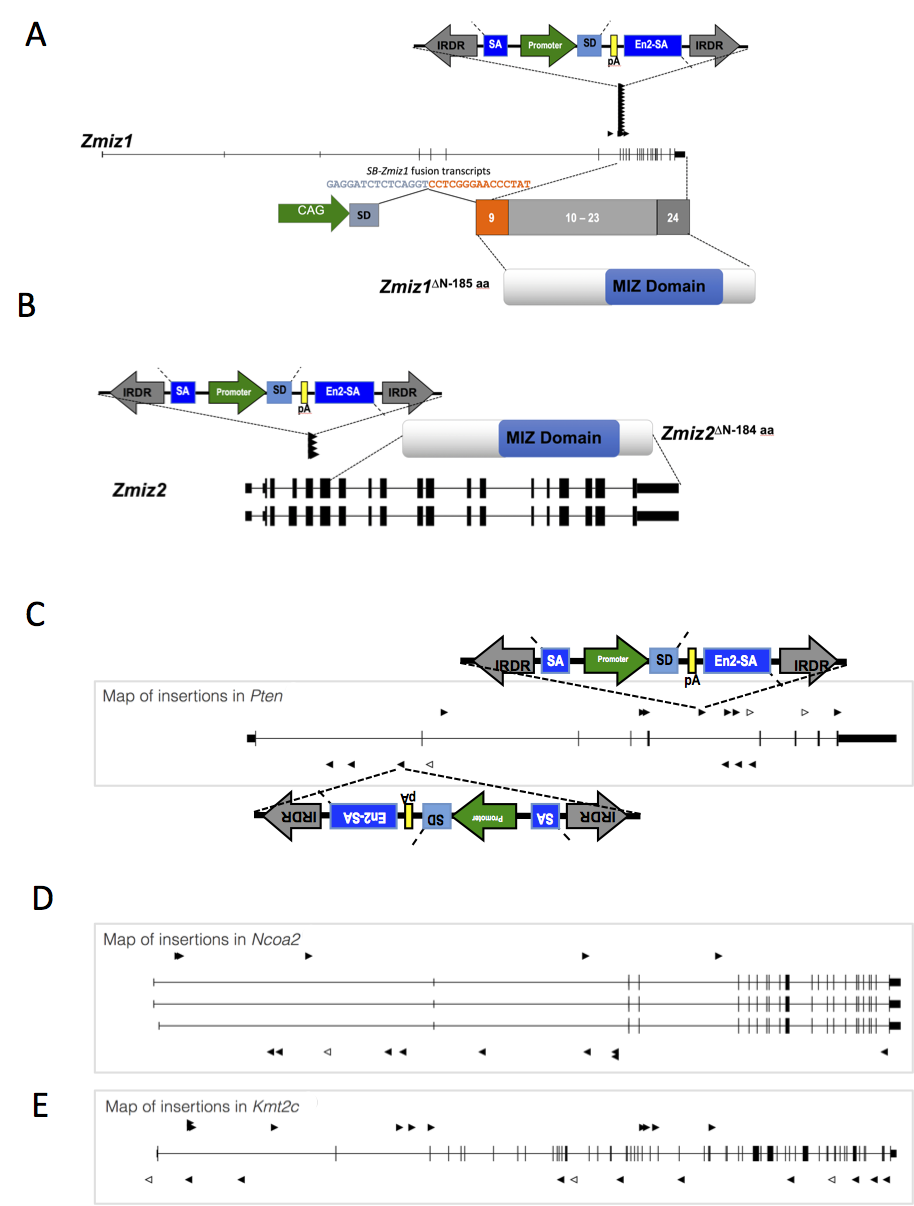

Supplement: S2 Fig — Recurrent TA-dinucleotide SB insertion events from individual cuSCC genomes reveal activating sense strand SB insertions in the locus of the paralogs Zmiz1 (A) or Zmiz2 (B). Chimeric fusion between the SB transposon splice donor (SD) and splice acceptor sites at exon 9 of Zmiz1 or exon 6 of Zmiz2 results in CAG-promoter driven transcription of a truncated mRNA predicted to encode N-terminally truncated Zmiz1ΔN-185 aa or Zmiz2ΔN-184 aa containing functional MIZ-type zinc finger domains and a Siz/PIAS RING finger (SP-RING). Representative SB insertion maps in cuSCC trunk drivers showing the locations of mapped SB insertions (triangles) predicted to inactivate tumor suppressors, shown in detail for Pten (C). Inactivating insertion maps for chromatin remodelers Ncoa2 (D) and Kmt2c (E). (TIFF) [file pgen.1009094.s002.tiff]

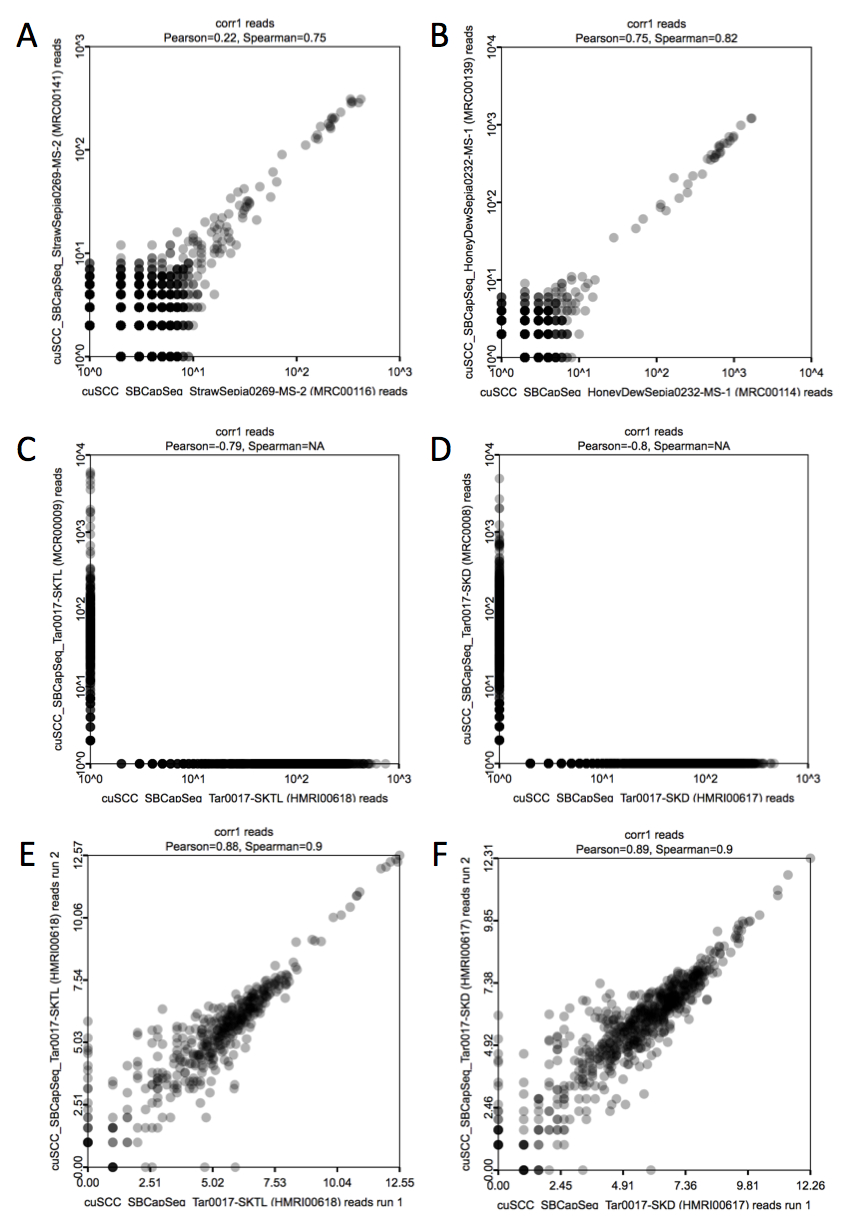

Supplement: S3 Fig — Representative plots of individual SB insertion sites based on read depth from two individual cuSCC genomes (A-B) and two unselected skin cell genomes from histologically normal skin (C-D) using genomic DNAs isolated from bulk tumor specimens from biological replicate libraries (independent library workflows applied to the same biological specimen isolate) comparing library 1 (x-axis) to library 2 (y-axis). Biological reproducibility of cuSCC specimen libraries is indicated by both Pearson’s and Spearman’s correlation metrics (A-B). No biological reproducibility of normal skin specimen libraries is observed (C-D) indicated by negative values for Pearson’s and Spearman’s correlation metrics and a failure to identify the same SB insertion sites. Individual SB insertion sites plotted by read depth from two technical replicates (the same library preparation) from two separate sequencing runs (E-F) confirms high reproducibility between individual libraries at high-to-moderate read depths, supported by both Pearson’s (r = 0.48) and Spearman’s (ρ = 0.49 or undetermined) correlation metrics. (TIFF) [file pgen.1009094.s003.tiff]

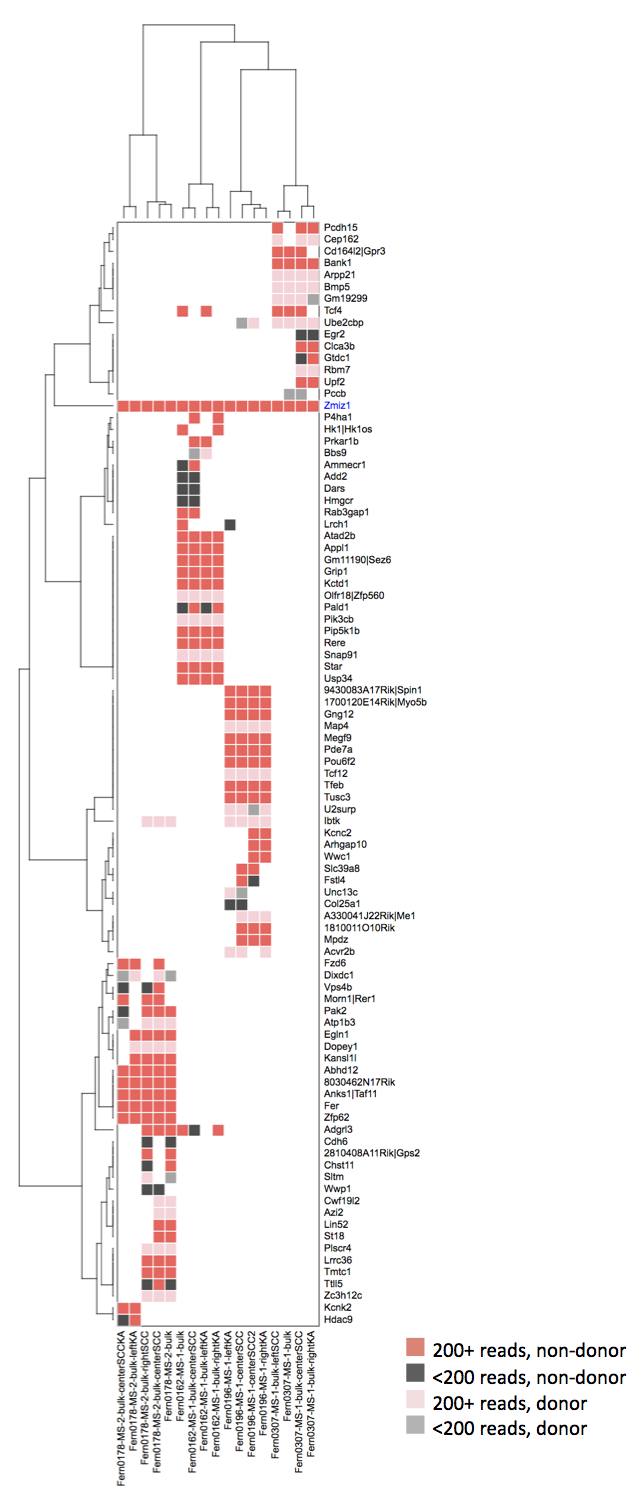

Supplement: S4 Fig — Hierarchical two-dimensional clustering (Hamming distance with the Ward method of agglomeration) of recurrent genic SBCapSeq insertion events from four skin masses containing distinct cuKA and cuSCC regions. Four distinct specimen (x-axis) and gene (y-axis) clades, each pertaining to a single bi-lesional mass, demonstrate clonal identities. Zmiz1 was the only gene recurrently mutated across all samples with high read depths, highlighted in blue text. (TIFF) [file pgen.1009094.s004.tiff]

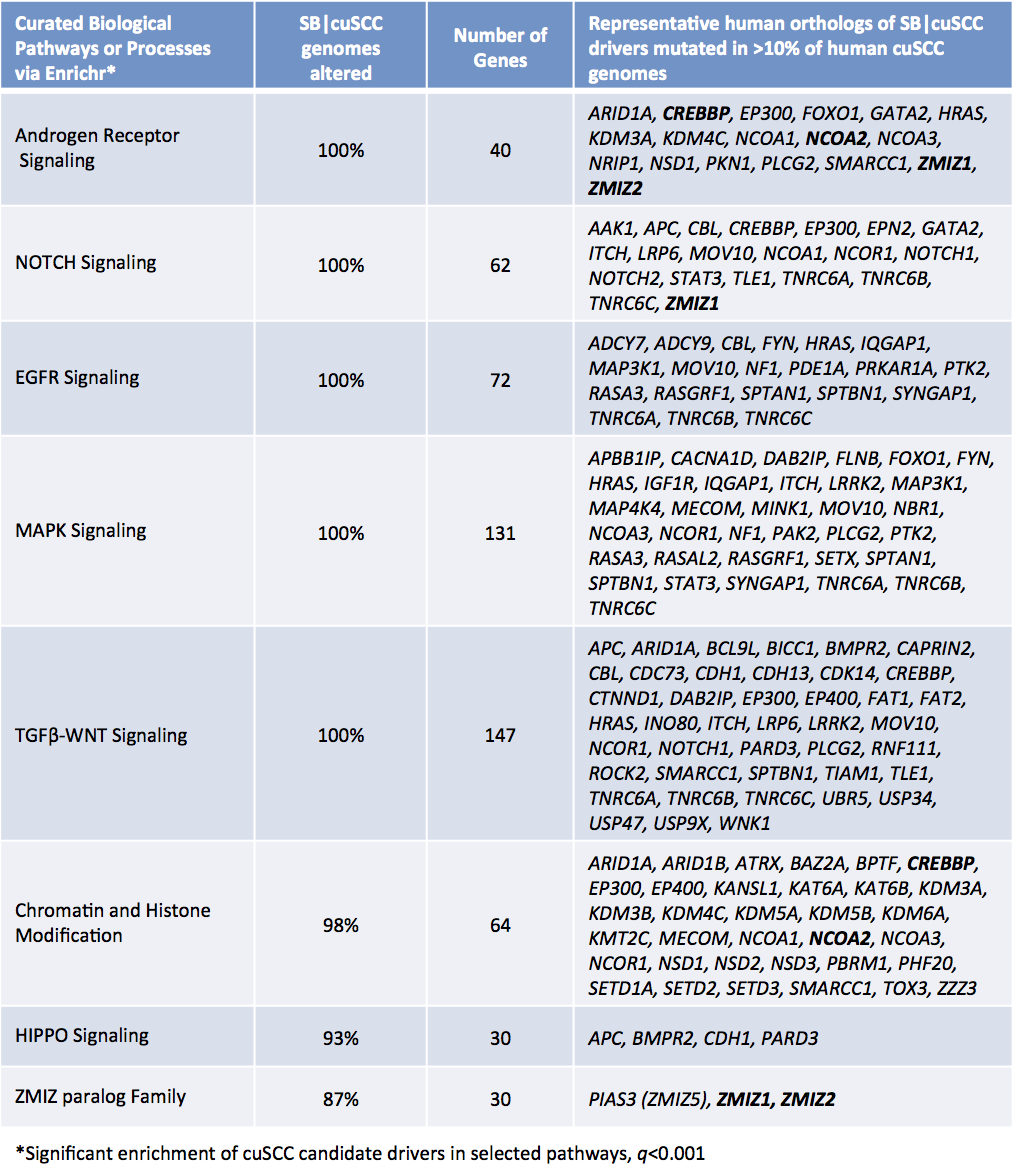

Supplement: S5 Fig — Table of significant pathways collated from pathway enrichment categories. (TIFF) [file pgen.1009094.s005.tiff]

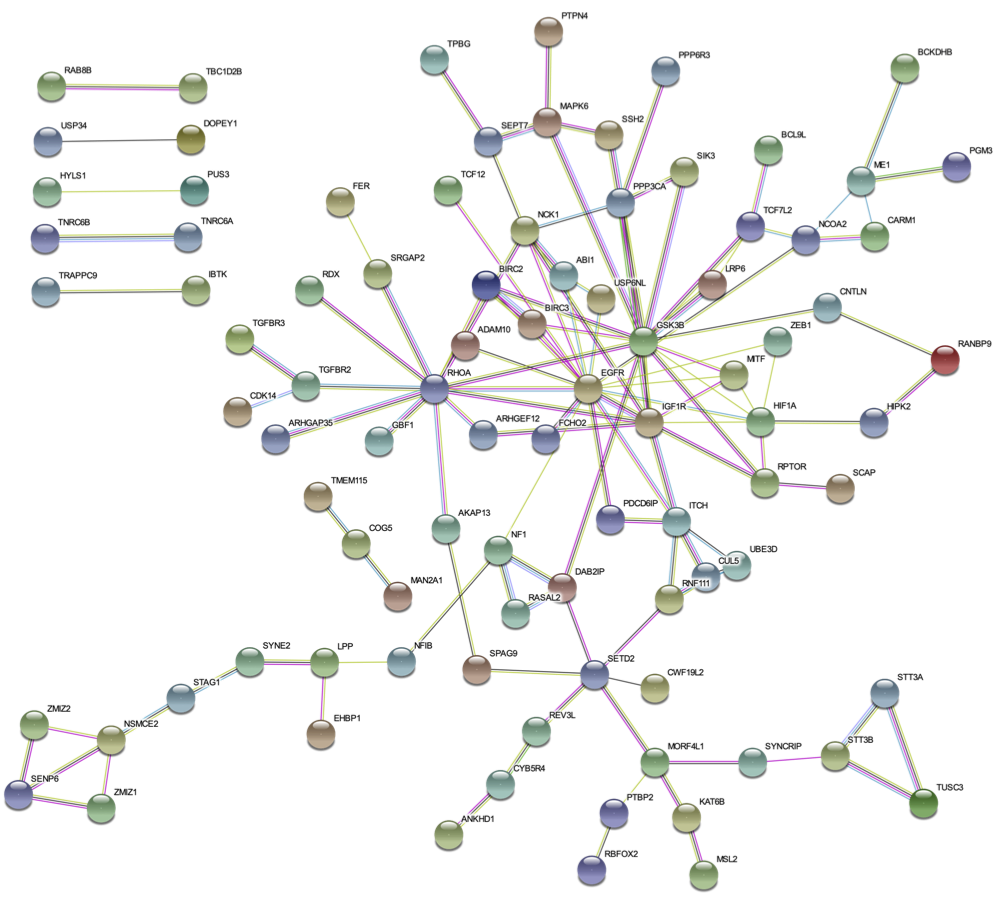

Supplement: S6 Fig — (A) The SB|cuSCC Trunk Driver (n = 84) network has significantly more known protein-protein interactions than expected by chance (P = 8.55 × 10−68.55e-06, STRING enrichment analysis; number of nodes: 144, number of edges: 124, expected number of edges: 82 (B) The SB|cuKA Trunk Driver (n = 62) network also has significantly more known protein-protein interactions than expected by chance (P = 6.93 × 10^−5^, STRING enrichment analysis; number of nodes: 37, number of edges: 14, expected number of edges: 4. (TIFF) [file pgen.1009094.s006.tiff]

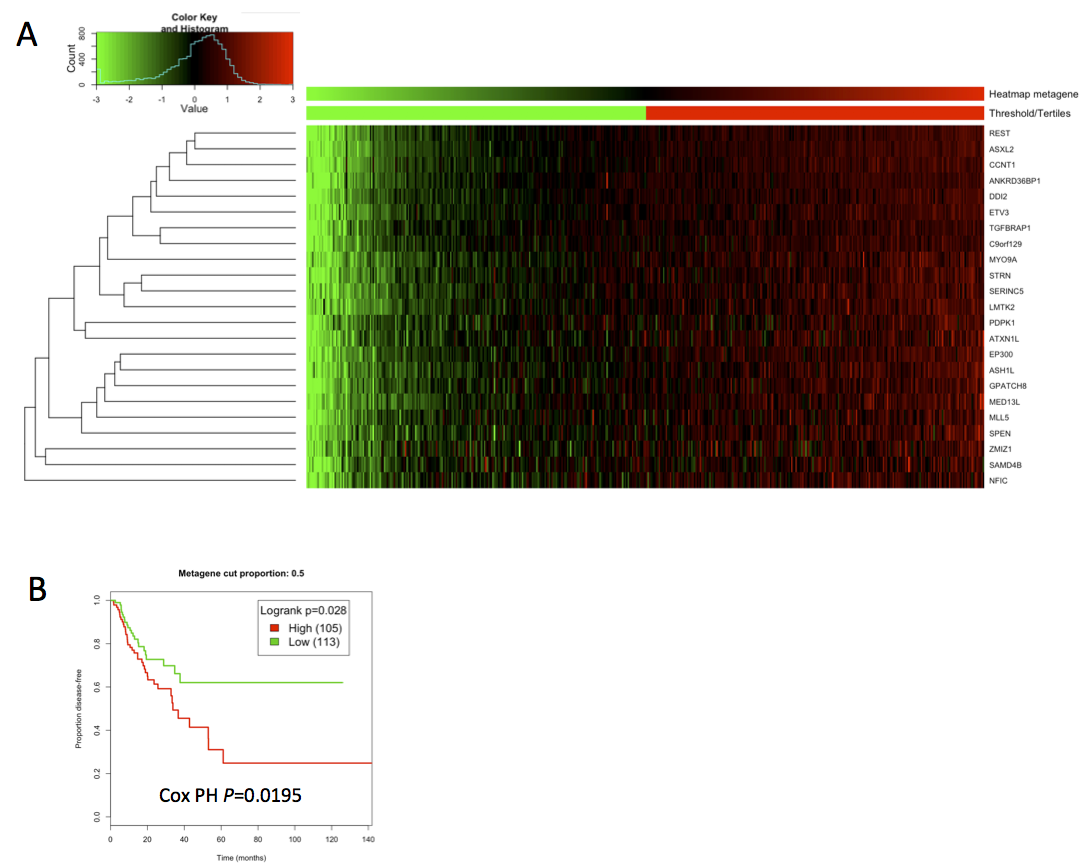

Supplement: S7 Fig — (A) ZMIZ1 metagene heatmap constructed using singular value decomposition of human hnSCC RNA-seq dataset from TCGA consisting of a 23-gene signature. Multivariate analysis of a gene signatures that correlates with the expression of ZMIZ1 in HNSCC. (B) Survival plots for patients with head and neck type SCC (hnSCC) based on the ZMIZ1–centric metagene. (TIFF) [file pgen.1009094.s007.tiff]

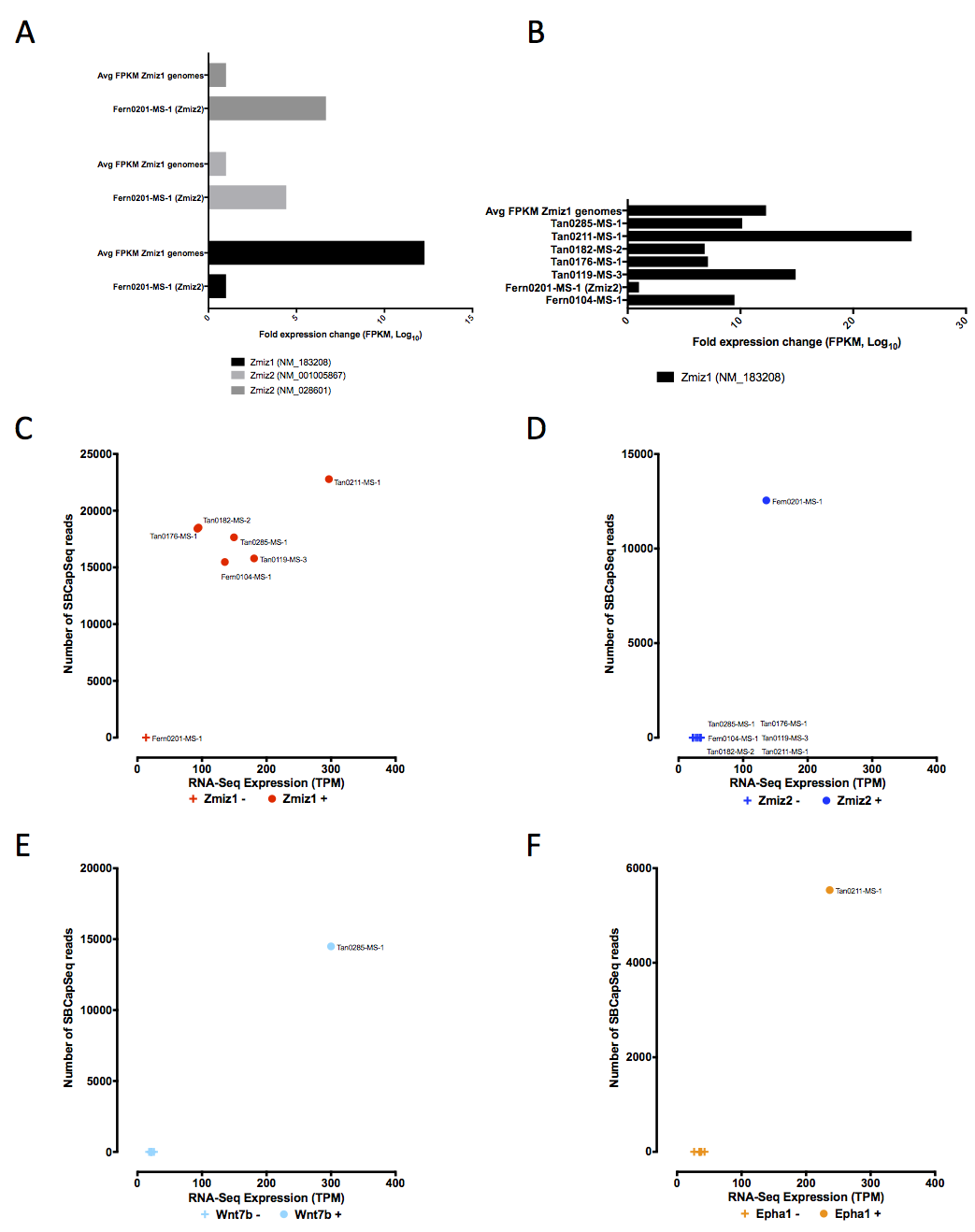

Supplement: S8 Fig — (A) Summary of Zmiz1 and Zmiz2 transcripts identified from bulk analysis of cuSCC cells by wtRNA-seq are represented as the log10 ratio of fragments per kilobase of transcript per million (FPKM) from the average of 6 genomes with SB insertions into Zmiz1 compared to the transcripts from the single genome with Zmiz2 SB insertion, defined by FPKM mapped reads from ribo-depleted RNA. (B) Transcripts identified from bulk analysis of cuSCC cells by wtRNA-seq are represented as the log10 ratio of SB insertion containing compared to wild-type transcripts, defined by fragments per kilobase of transcript per million mapped reads from ribo-depleted. (C-F) Individual panels from Fig 5E: multifold induction of gene expression in cuSCC masses with (+) high read depth activating SB insertion events among 4 candidate oncogenic drivers compared with normal gene expression levels in cuSCC tumors without (–) SB insertions. (TIFF) [file pgen.1009094.s008.tiff]

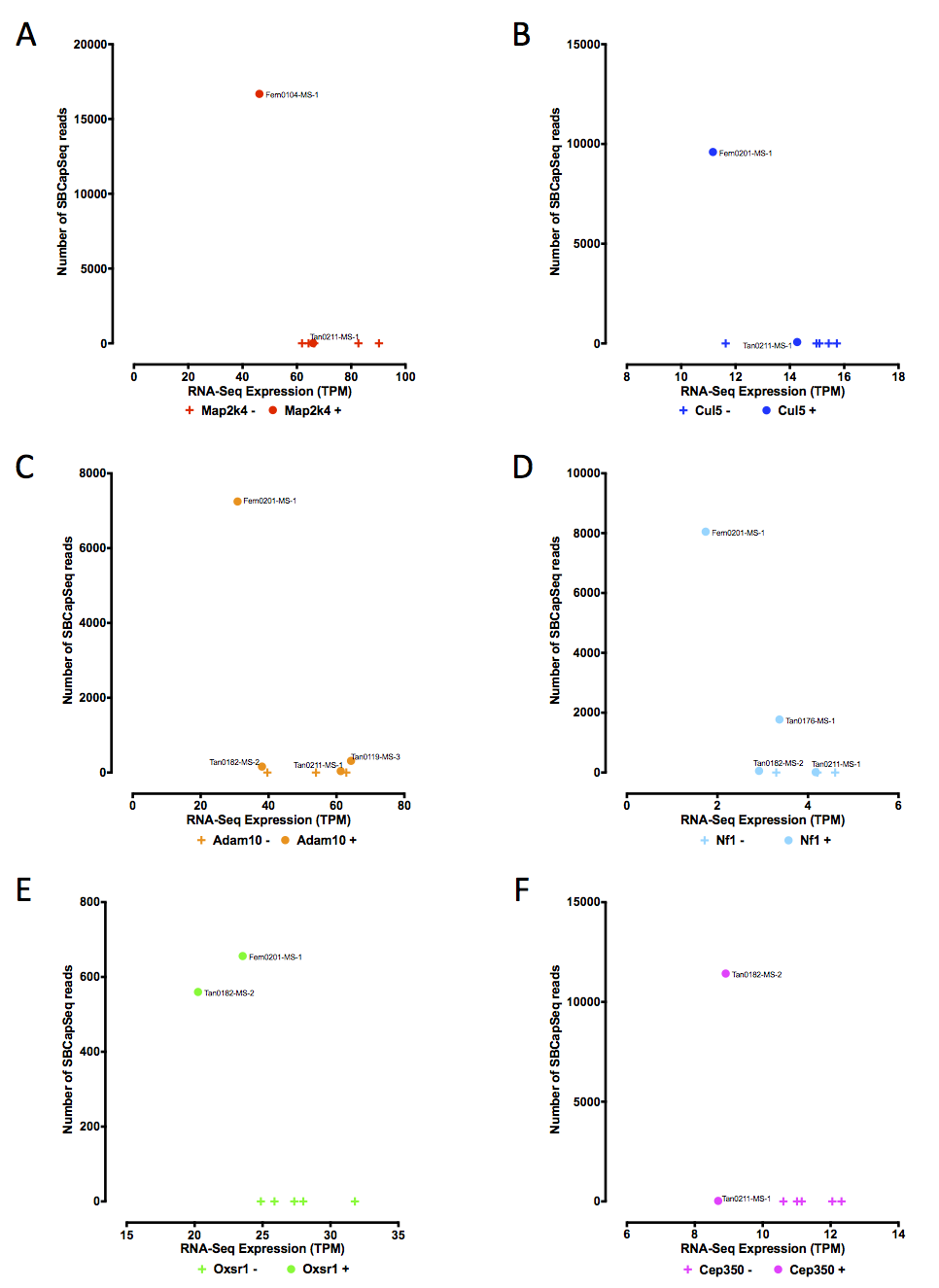

Supplement: S9 Fig — (A-F) Individual panels from Fig 5F: reduced gene expression in cuSCC masses with (+) high read depth inactivating SB insertion events among 6 candidate tumor suppressor drivers compared with normal gene expression levels in cuSCC tumors without (–) SB insertions. (TIFF) [file pgen.1009094.s009.tiff]

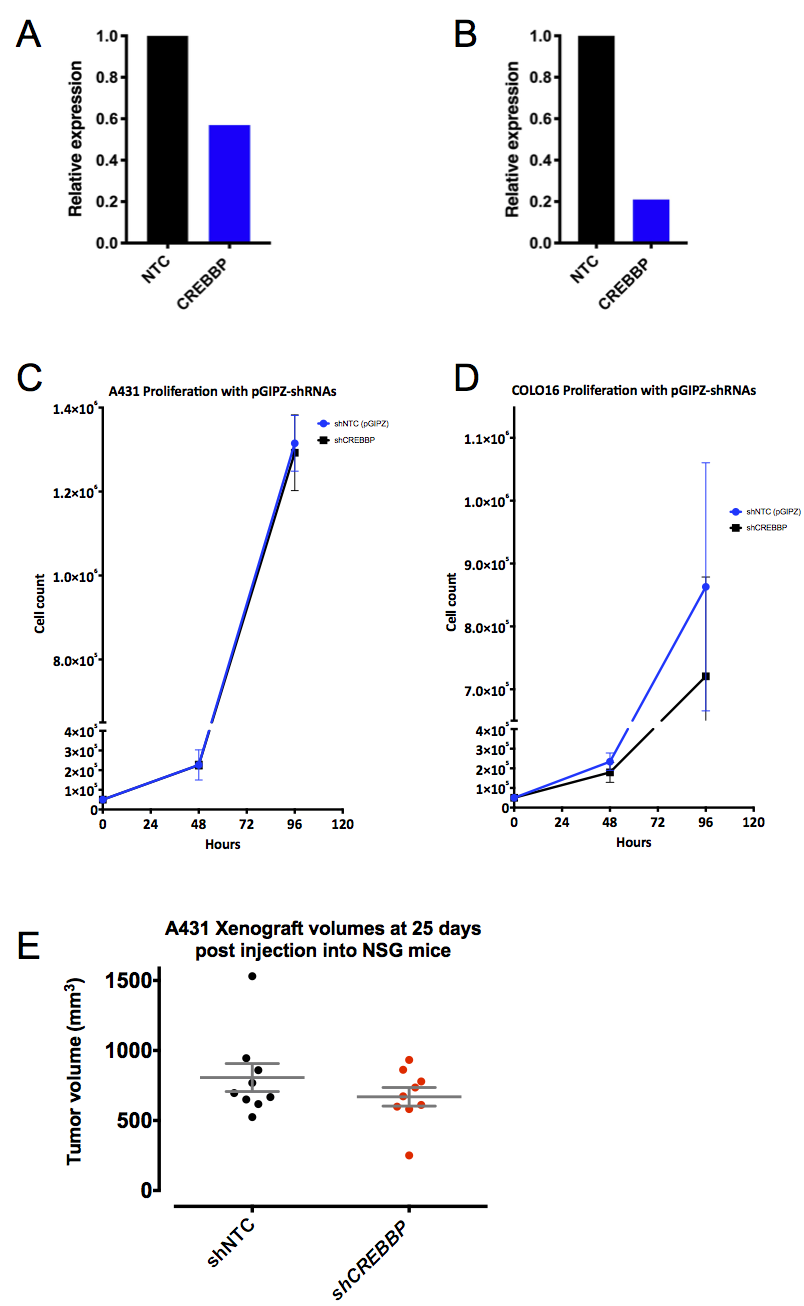

Supplement: S10 Fig — (A) 96-hour proliferation assay (n = 3, error bars SEM). (B) In vivo xenograft assay of COLO16 shNTC or shCREBBP into NSG immunodeficient mice (n = 9 per condition). (TIFF) [file pgen.1009094.s010.tiff]

A

## A431 @ 25 days

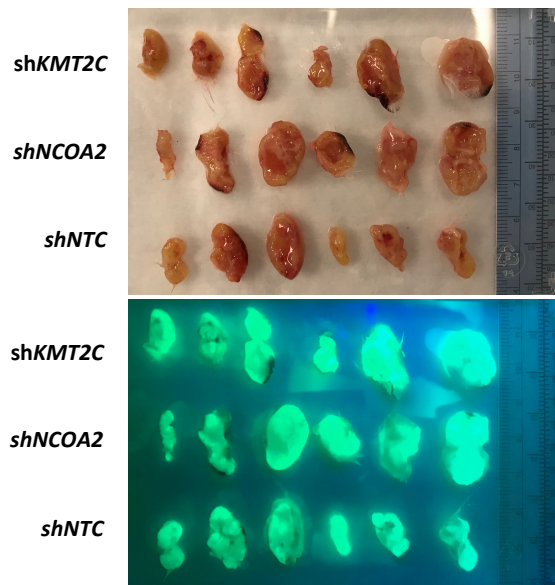

B

## A431 @ 25 days

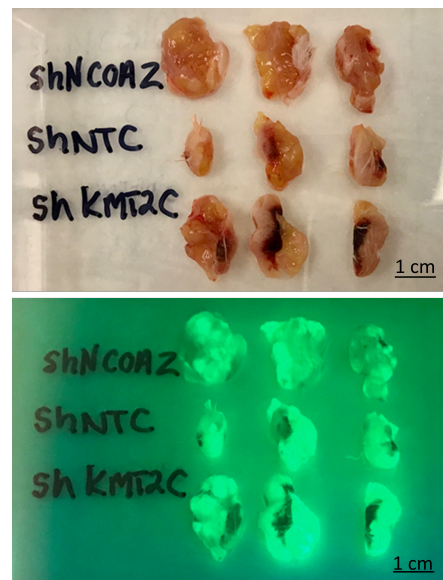

C

## COLO16 @ 18 days

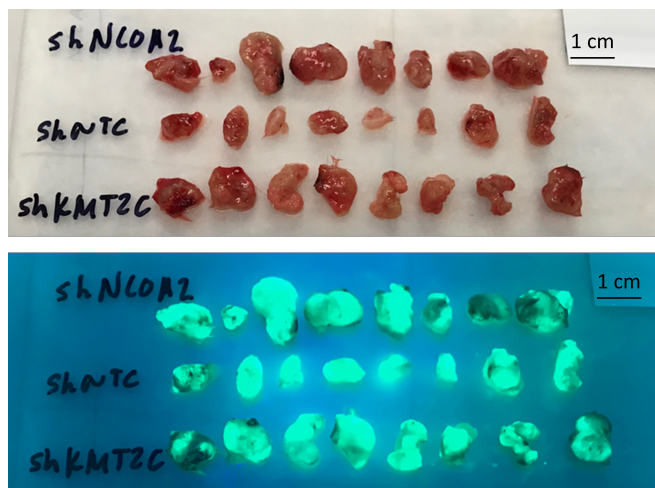

D

## SCC13 @ 49 days

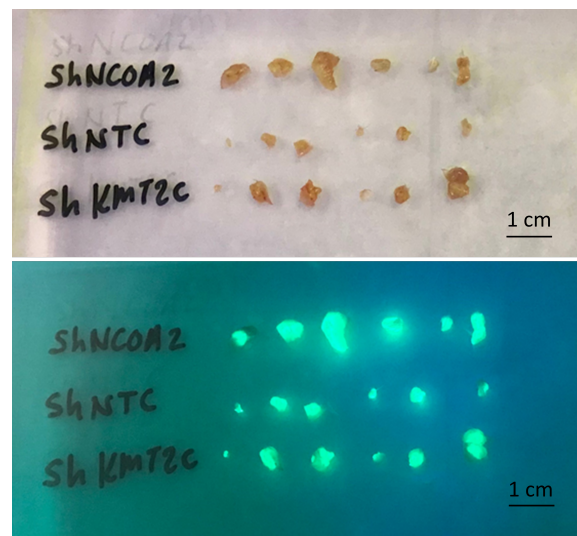

Supplement: S11 Fig — Xenografts of cuSCC human cell lines A431, COLO16 and SCC13 with stable lentiviral constructs expressing shRNAs directed knockdown of target drivers KMT2C (shKMT2C) or NCOA2 (shNCOA2) relative to a non-targeting control (shNTC) grown in NSG immunodeficient mice. (PDF) [file pgen.1009094.s011.pdf]

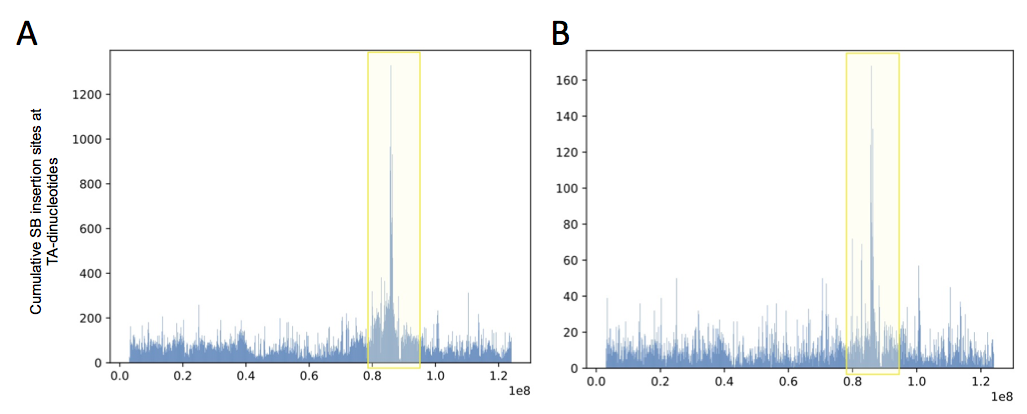

Supplement: S12 Fig — Cumulative SB insertion sites at TA-dinucleotides from (A) 454_Splink and (B) Ion_SBCapSeq datasets showing the likely TG.12740 donor site at 87,000,000 bp and the exclusion region (yellow box) between 79,000,000 and 95,000,000 bp applied when SB Driver Analysis was run on chromosome 9. The 81 genes between Filip1 at chr9:79663368–79825689 and Tfdp2 at chr9:96096693–96224065 were excluded from SB Driver Analysis. The complete list of 81 genes excluded from SB Driver Analysis from tumors harboring the SB T2/Onc3 TG.12740 donor allele: 1190002N15Rik, 1700034K08Rik, 1700057G04Rik, 1700065D16Rik, 4930524O08Rik, 4930554C24Rik, 4933400C23Rik, 9330159M07Rik, 9430037G07Rik, A330041J22Rik, Adamts7, AF529169, Ankrd34c, Atr, B430319G15Rik, Bckdhb, Bcl2a1a, Bcl2a1b, Bcl2a1d, Cep162, Chst2, Ctsh, Cyb5r4, D430036J16Rik, Dopey1, Elovl4, Fam46a, Gk5, Hmgn3, Htr1b, Ibtk, Impg1, Irak1bp1, Lca5, Me1, Mei4, Mir184, Mir6386, Mir7656, Morf4l1, Mrap2, Mthfs, Mthfsl, Myo6, Nt5e, Paqr9, Pcolce2, Pgm3, Phip, Plod2, Pls1, Plscr1, Plscr2, Plscr4, Plscr5, Prss35, Rasgrf1, Ripply2, Rwdd2a, Senp6, Sh3bgrl2, Slc9a9, Snap91, Snhg5, Snx14, Syncrip, Tbc1d2b, Tbx18, Tmed3, Tpbg, Trim43a, Trim43b, Trim43c, Trpc1, Ttk, U2surp, Ube2cbp, Xrn1, Zfp949, Zic1, Zic4. (TIFF) [file pgen.1009094.s012.tiff]
