# Supplementary material for: Transposon mutagenesis identifies cooperating genetic drivers during keratinocyte transformation and cutaneous squamous cell carcinoma progression
Source: PLoS Genet. 2021 Aug 16;17(8):e1009094. doi: 10.1371/journal.pgen.1009094 (PMC8389471; doi:10.1371/journal.pgen.1009094)
Supplement: S1 Text — (DOCX) [file pgen.1009094.s033.docx]

**Supplementary Note:**

**Oncogenomic comparisons between SB candidate Trunk driver genes and their direct orthologs in human Cancer Gene Census.**

Contingency Tables and Venn diagrams comparing Cancer Gene Census (GRCh38 COSMICv86) driver enrichment with various cuSCC Trunk and Progression Driver cohort source data are available the NIH-Figshare data repository (https://doi.org/10.35092/yhjc.11441130.v1, ref [1]).

**Pyrosequencing analysis of SB-driven keratinocyte cancer models**

To investigate SB-driven keratinocyte transformation and cuSCC progression in wild type and Trp53 mutant mice, we sequenced SB insertions sites using the 454_splink method [2] from 83 individual skin masses isolated from 65 SB|Onc3 mice, that were histologically confirmed as cutaneous squamous cell carcinoma (cuSCC). Using gene-guided SB Driver Analysis statistical framework [3], we identified 31,266 non-redundant transposon insertion sites from the individual or combined analysis of Trp53 mutant and wild type cuSCC cohorts which were used to statistically define 428 candidate cancer drivers (CCDs). Nearly all CCDs were identified across all cohorts, suggesting that their role in driving cuSCC is independent of *Trp53* mutation. For this reason, we focused subsequent data analysis on the superset consisting of all SB|cuSCC masses with all *Trp53* alleles. Previous studies suggest that most CCDs identified by SB mutagenesis function during tumor progression rather than tumor initiation [4]. We previously reported a workflow that allow us to distinguish initiating and progression drivers within bulk tumor and single cell sequencing in an SB-driven myeloid leukemia model [2]. Stringent filtering of the SB insertions to include only events with the highest sequencing read depths defines trunk driver alterations that are predicted to be under positive-selection within the clonally expanding tumors, where they participate in either initiation or early progression of cellular transformation [2, 4].

To identify CCDs that drive keratinocyte transformation, we selected the SB insertion sites with the highest number of sequencing reads to define trunk drivers, reasoning that tumor initiating insertions would have among the highest frequency in tumor cells. Using SB insertions sites by the 454_splink method, we identified 142 trunk drivers of initiation and/or early progression of keratinocyte transformation *in vivo* (subsequently referred to as trunk drivers). SB insertion maps for representative cuSCC trunk drivers show the locations of mapped SB insertions and define candidate cancer drivers as putative proto-oncogenes or tumor suppressor candidate cuSCC driver genes.

In a previously published study, a surprising insight into genotype-phenotype relationships during keratinocyte transformation was found by comparing the sequencing of early-stage cuKA masses that arose spontaneously in a breast cancer cohort consisting of quadruple heterozygous mice carrying alleles for K5-Cre, Pten-floxed, Onc3(TG.12740), ^LSL^ SBase to phenotypically indistinguishable cuKA masses observed in the SB|P53 cohorts [5,6]. We investigated keratinocyte transformation using keratinocyte-specific SB mutagenesis in *Pten*-sensitized mice. The screen originally was designed to activate an inducible allele of the SB transposase (Rosa26-LSL-SB11) using *Keratin5*-Cre (K5-Cre, Tg(KRT5-cre)5132Jlj, MGI:3050065) [7] to drive SB insertional mutagenesis in stratified epithelium of the mammary ducts in a haploinsufficient *Pten^floxed/+^* sensitized background [5]. However, K5 also induces cre expression in cells of the basal epidermis. Using a two-generation mating scheme, we first introduced K5-Cre onto the background of a Cre-LoxP inducible *Pten* mutant strain, *Pten^CKO/+^* (*Pten*^tm1Hwu^ (MGI:2156086)). Compound heterozygous progeny (K5-Cre; *Pten^CKO/+^*) were mated with double homozygous SB strains containing either a high-copy (T2/Onc2-TG.6113) or a low-copy (T2/Onc3-TG.12740) transposon donor concatemer and the inducible SB transposase (Rosa26-LSL-SBase or SBase^LSL^) to generate experimental and control cohorts [13]. Cutaneous keratoacanthomas (cuKA) were diagnosed from routine H&E sections and found to occur in 43% (13 of 30) of quadruple mutant mice, either K5-Cre; *Pten^CKO/+^*; TG.6113; SBase^LSL^ or K5-Cre; *Pten^CKO/+^*; TG.12740; SBase^LSL^. Four of the mice with cuKA also developed a breast tumor and none of the mice in the control groups developed skin masses before reaching a predetermined experimental end-point at 450 days of age.

To identify SB drivers of cuKA in *Pten*-sensitized mice, we used 45 flash frozen cuKA specimens from 13 quadruple mutant mice collected at necropsy to create genomic DNA and sequenced using the 454-Splink method to sequencing SB insertion events. Using the 454 sequencing data, we performed gene-guided SB Driver Analysis [3] to define 302 statistically significant candidate cancer drivers and 29 Trunk drivers. Our top ranked genes were *Chuk* (*IKKA*/*IKK1*), *Notch1*, and *Kctd15*. All genes were found to have inactivating patterns of SB insertion events into each of their gene-coding regions, strongly suggesting that cooperating tumor suppressors drive early keratinocyte transformation and cuKA initiation *in vivo*. However, three genomes lacking *Chuk* insertions were found to have activating SB insertions into *Zmiz1* that were identical to those observed in the cuSCC cohorts. This may suggest that the altered function(s) of *Chuk* in cuKAs may be overlapping with those of the SB-induced *Zmiz1*^ΔN185^ [8] allele. In keratinocytes with SB mobilization and heterozygous loss of *Pten*, activating SB insertions into *Zmiz1, Zmiz2,* and *Mamld1* were rarely, if ever, observed. Instead, inactivating insertions into *Chuk* were statistically over-represented and, when they occurred, were mutually exclusive with *Zmiz1* or *Mamld1* insertions, suggesting they may be within the same genetic pathway. Surprisingly, SBCapSeq analysis of normal skin from 9 separate mice with the same quadruple heterozygous genotype revealed many low-read depth insertions into the *Pten* locus, but no insertions in *Chuk*, suggesting that selection for *Chuk* inactivation may be dependent upon haploinsufficient loss of *Pten*. In addition, using the datasets within the recently published SBCDDB [9], we found statistically significant evidence for co-occurring loss of *Pten* (either via SB insertion or by breeding in a heterozygous null allele) and SB inactivation of *Chuk* across solid tumor models (Fisher's exact test, *P*=2.36 × 10^-7^). Statistical significance for co-occurring mutations in *Pten* and *Chuk* in solid cancers persisted even after removing all cuKA tumors from the analysis (Fisher's exact test, *P*=3.78 × 10^-16^), suggesting this relationship may be generally observed in non-keratinocyte tumor lineages. Remarkably, querying for co-occurrence of alterations in *PTEN* and *CHUK* via cBioPortal, across 41,834 patient samples from 170 studies, revealed significant co-occurrence for these two genes in human cancers (Chi-square with Yates correction, χ^2^=406, *P*<0.001). Collectively, these data suggest that coordinated loss of both *Pten* and *Chuk* may have a role in initiating and progressing oncogenic transformation in keratinocyte as well as non-keratinocyte lineages. Given the recurring inactivating SB insertions in *Chuk* and activating *Zmiz1* observed in our *Pten*-loss sensitized mice, it is curious that a recent separate *Pten*-sensitized SB screen for cooperating TSGs failed to identify *Chuk* or *Zmiz1* entirely [10]. One possibility for this difference is that the reported screen used a single-copy SB allele, while our screen used multi-copy SB alleles with multiple independent genic insertion events per tumor, and the lack of *Chuk* or *Zmiz1* insertion events may suggest that positive selection for variant *Chuk* or *Zmiz1* alleles in haploinsufficient *Pten*-mutant skin cells may require one or more additional cooperating drivers. Finally, a recent meta-analysis of human cuSCC genomes revealed statistically significant somatic mutation burdens in *CHUK* as an underappreciated candidate cancer gene [11], further supporting that alterations in *CHUK* may be an important driver of keratinocyte transformation.

Roche 454 sequencing source data available the NIH-Figshare data repository (<https://doi.org/10.35092/yhjc.11441130.v1>, ref [1]).

**References:**

1. Mann MB, Aiderus A, Newberg JY, Guzman-Rojas L, Contreras-Sandoval AM, Meshey AL, et al. Supplementary Tables1-43 for 'Transposon mutagenesis identifies cooperating genetic drivers during keratinocyte transformation and cutaneous squamous cell carcinoma progression'2019. The NIH Figshare Archive. Dataset. https://doi.org/10.35092/yhjc.11441130.v1.

2. Mann KM, Newberg JY, Black MA, Jones DJ, Amaya-Manzanares F, Guzman-Rojas L, et al. Analyzing tumor heterogeneity and driver genes in single myeloid leukemia cells with SBCapSeq. *Nat* *Biotechnol*. 2016;34(9):962-72.

3. Newberg JY, Black MA, Jenkins NA, Copeland NG, Mann KM, Mann MB. SB Driver Analysis: a *Sleeping* *Beauty* cancer driver analysis framework for identifying and prioritizing experimentally actionable oncogenes and tumor suppressors. *Nucleic Acids Res*. 2018.

4. Mann MB, Black MA, Jones DJ, Ward JM, Yew CC, Newberg JY, et al. Transposon mutagenesis identifies genetic drivers of Braf(V600E) melanoma. *Nat Genet*. 2015;47(5):486-95.

5. Rangel R, Lee SC, Hon-Kim Ban K, Guzman-Rojas L, Mann MB, Newberg JY, et al. Transposon mutagenesis identifies genes that cooperate with mutant Pten in breast cancer progression. *Proc Natl Acad Sci U S A*. 2016;113(48):E7749-E58.

6. Stransky N, Egloff AM, Tward AD, Kostic AD, Cibulskis K, Sivachenko A, et al. The mutational landscape of head and neck squamous cell carcinoma. *Science*. 2011;333(6046):1157-60.

7. Ramirez A, Page A, Gandarillas A, Zanet J, Pibre S, Vidal M, et al. A keratin K5Cre transgenic line appropriate for tissue-specific or generalized Cre-mediated recombination. *Genesis*. 2004;39(1):52-7.

8. Rogers LM, Riordan JD, Swick BL, Meyerholz DK, Dupuy AJ. Ectopic expression of *Zmiz1* induces cutaneous squamous cell malignancies in a mouse model of cancer. J Invest Dermatol. 2013;133(7):1863-9.

9. Newberg JY, Mann KM, Mann MB, Jenkins NA, Copeland NG. SBCDDB: *Sleeping* *Beauty* Cancer Driver Database for gene discovery in mouse models of human cancers. *Nucleic Acids Res*. 2017.

10. de la Rosa J, Weber J, Friedrich MJ, Li Y, Rad L, Ponstingl H, et al. A single-copy *Sleeping* *Beauty* transposon mutagenesis screen identifies new PTEN-cooperating tumor suppressor genes. *Nat Genet*. 2017;49(5):730-41.

11. Chang D and Shain AH. The landscape of driver mutations in cutaneous squamous cell carcinoma. *NPJ Genom Med. 2021 Jul 16;6(1):61.*
